# *Mycobacterium abscessus* alkyl hydroperoxide reductase C promotes cell invasion by binding to tetraspanin CD81

**DOI:** 10.1016/j.isci.2023.106042

**Published:** 2023-01-25

**Authors:** Jona Karam, Fabien P. Blanchet, Éric Vivès, Prisca Boisguérin, Yves-Marie Boudehen, Laurent Kremer, Wassim Daher

**Affiliations:** 1 Centre National de la Recherche Scientifique UMR 9004, Institut de Recherche en Infectiologie de Montpellier (IRIM), Université de Montpellier, 1919 Route de Mende, 34293 Montpellier, France; 2 PhyMedExp, University of Montpellier, INSERM U1046, CNRS UMR, 9214 Montpellier, France; 3 INSERM, IRIM, 34293 Montpellier, France

**Keywords:** Immunology, Microbiology, Bacteriology

## Abstract

*Mycobacterium abscessus* (*Mab*) is an increasingly recognized pulmonary pathogen. How *Mab* is internalized by macrophages and establishes infection remains unknown. Here, we show that *Mab* uptake is significantly reduced in macrophages pre-incubated with neutralizing anti-CD81 antibodies or in cells in which the large extracellular loop (LEL) of CD81 has been deleted. Saturation of *Mab* with either soluble GST-CD81-LEL or CD81-LEL-derived peptides also diminished internalization of the bacilli. The mycobacterial alkyl hydroperoxide reductase C (AhpC) was unveiled as a major interactant of CD81-LEL. Pre-exposure of macrophages with soluble AhpC inhibited mycobacterial uptake whereas overexpression of AhpC in *Mab* enhanced its internalization. Importantly, pre-incubation of macrophages with anti-CD81-LEL antibodies inhibited phagocytosis of AhpC-coated beads, indicating that AhpC is a direct interactant of CD81-LEL. Conditional depletion of AhpC in *Mab* correlated with decreased internalization of *Mab*. These compelling data unravel a previously unexplored role for CD81/AhpC to promote uptake of pathogenic mycobacteria by host cells.

## Highlights

• Neutralization of CD81 restricts uptake of *Mycobacterium abscessus* into macrophages
• Knock-down expression of AhpC in *M. abscessus* reduces mycobacterial uptake
• Anti-CD81 antibodies reduce the phagocytosis of AhpC-coated beads
• AhpC binds to CD81 to facilitate *M. abscessus/*macrophage early interactions

## Graphical abstract

**Figure:**
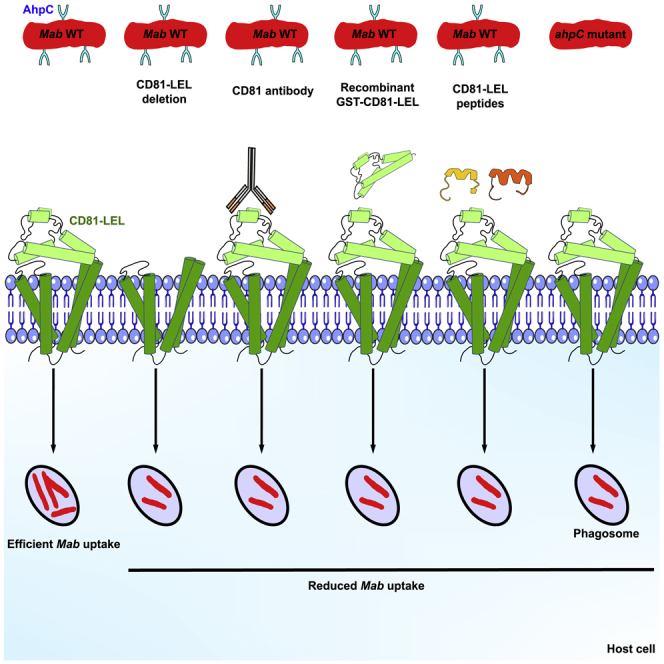

## Introduction

Non-tuberculous mycobacteria (NTM) are widely distributed in water pipes and soil environment.^1^ They are acquired through the environment and can also be transmitted by aerosolization and subsequent inhalation from one person to another, particularly in immunocompromised individuals or in cystic fibrosis patients.^2^ NTM lung disease is becoming more common and accounts for the majority of clinical cases of NTM infection.^3^ Despite that NTM infection can affect practically every organ, pulmonary infection remains the most common clinical manifestation.^4^ NTM are split into two groups based on their growth rate: fast-growing and slow-growing.^5^ Among these, *M. abscessus* (*Mab*) represents the most virulent fast-growing species, responsible for pulmonary and extrapulmonary infections, primarily skin infections, and is commonly associated with nosocomial infections.^6^ *Mab* can infect, survive, and replicate in a variety of cell types.^7^ Endothelial epithelial cells, fibroblasts, antigen-presenting cells such as macrophages, dendritic cells, and neutrophils have been identified as potential *Mab* host cells.^8^^,^^9^^,^^10^^,^^11^^,^^12^^,^^13^^,^^14^ *Mab* is found in macrophages where it manages to multiply, in contrast to other fast-growing NTM or non-pathogenic species, which are naturally cleared by macrophages.^15^^,^^16^^,^^17^ The infection process begins when bacterial products, known as pathogen-associated molecular patterns (PAMP), engage with pattern recognition receptor (PRR), such as Toll-like receptors (TLR) or C-type lectins present in the plasma membrane.^18^ PAMP binding activates either permissive or restrictive signaling pathways, which is followed by phagocytosis.^19^ Phagocytosis of mycobacteria usually results in the formation of phagosomes, which mature and eventually fuse with lysosomes to form phagolysosomes, where microbial destruction occurs.^20^ *Mab* not only survives, but also replicates within macrophages, limiting phagosome maturation and, as a result, lysosome fusion.^15^^,^^21^ Thus, understanding the interactions between the host and the pathogen is crucial for the development of host-directed treatments that can prevent bacterial uptake. However, PRR that contribute to the early interaction between *Mab* and the macrophage are poorly characterized.

Tetraspanins belong to a superfamily of glycoproteins, which span the membrane four times and possess a small (SEL) and a large extracellular loop (LEL).^22^ These proteins display a wide panel of cellular functions (adhesion, motility, and fusion) and are related to infection by a variety of human pathogens, including viruses, parasites and bacteria.^22^ Humans possess 33 tetraspanins, with most cells expressing simultaneously multiple members of this family of proteins.^23^ The LEL domains show the largest sequence diversity and are involved in member-specific functions.^24^ In addition, LEL domains can be recombinantly produced with a conserved biological activity. Tetraspanin actions are based on their capacity to connect with other molecules present in the membrane, generating functional assemblies known as tetraspanin-enriched microdomains (TEM).^25^ There is mounting evidence that viruses, parasites, and bacteria can bind to and infiltrate host cells using various tetraspanins.^22^ CD81 is a hepatitis C virus receptor^26^ that can also influence HIV-1 membrane fusion and viral clustering at the viral synapse.^27^ It is also necessary for the infection of hepatocytes by *Plasmodium*.^28^ Following infection of epithelial cells by Listeria, CD81 is attracted to the site of bacterial entrance.^29^ However, a possible CD81-dependent mechanism underlying mycobacterial entrance into macrophages or lung epithelial cells remains yet to be established.

The goal of this study consisted to stimulate the discovery of a hitherto unknown ligand-receptor interaction that mediates *Mab* infection in macrophages and lung epithelial cells. We evaluated whether CD81 may represent a PRR that favors the internalization of *Mab* inside human macrophages and lung epithelial cells. The combination of biochemical and mass spectrometry techniques led to the identification of AhpC as a strong mycobacterial candidate acting as a CD81-specific PAMP. Subsequent genetic studies and cellular biology approaches were conducted to confirm and validate the importance of the CD81/AhpC interaction in promoting the recognition and internalization of *Mab* by macrophages.

## Results

### CD81 is required for invasion of macrophages by *Mab*

**Figure 1:**
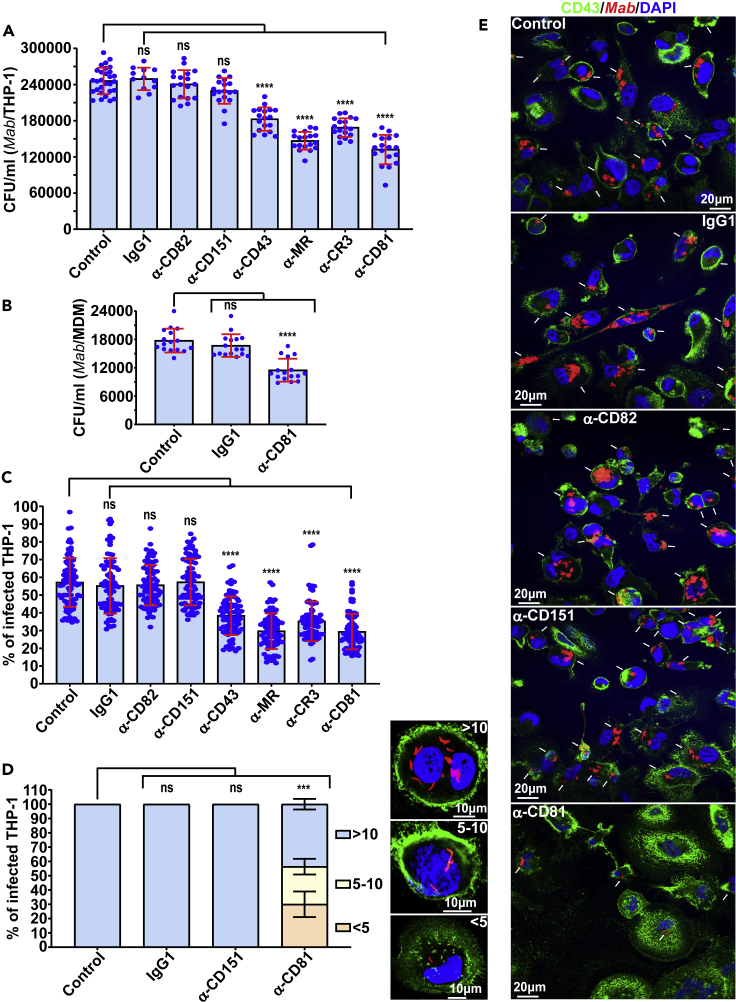
Internalization of *Mab* by macrophages requires tetraspanin CD81 (A and B) Uptake of *Mab* is reduced in THP-1 macrophages (A) and primary human macrophages (B) pre-treated with neutralizing antibodies raised against CD43, MR, CR3 or CD81. Macrophages were lysed with 1% Triton X-100 and then successive dilutions of the different suspensions were prepared and plated on LB agar before CFU counting. Data are mean values ±SD for six (A) or four (B) independent experiments, each time in triplicate (A) or quadruplicate (B), n = 18 (A) or 16 (B). One-tailed Tukey’s test: ns, non-significant > 0.05, ∗∗∗∗p < 0.0001. (C) Blocking CD43 or MR or CR3 or CD81 receptors with neutralizing antibodies significantly reduces the percentage of *Mab*-infected THP-1 macrophages. Quantification of the percentage of host cells containing bacilli was performed using an epifluorescence microscope. Images were acquired focusing on the combined signals (CD43 in green and *Mab* in red) and scoring for the presence or absence of bacilli in macrophages using ImageJ. Data are mean values ±SD for four independent experiments (n = 80 fields). One-tailed Tukey’s test: ns, non-significant > 0.05, ∗∗∗p < 0.001. (D) Percentage of *Mab*-infected THP-1 macrophage categories after pre-treatment with neutralizing antibodies raised against CD151 or CD81. The images illustrate a macrophage < 5 bacilli, 5 to 10 bacilli or > 10 bacilli (upper right). The nuclei were stained with DAPI (blue) and the surface of macrophages was stained with anti-CD43 antibodies (green). Values are means ± SD for three independent experiments performed each time in triplicate (n = 900 infected macrophages). One-tailed non-paired t-test: ns, non-significant > 0.05, ∗∗∗p < 0.001. (E) Five immunofluorescent fields were taken at 3 h post-infection using a confocal microscope (40× magnification), showing the macrophages infected with the *Mab* (in red) following pre-treatment with indicated antibodies. The nuclei are in blue and the CD43 protein associated with the plasma membrane of macrophages is in green. White arrows indicate *Mab* inside the cells.

Tetraspanin CD81 is expressed on the host cell surface and is identified as a receptor for the hepatitis C virus (HCV) E2 envelope glycoprotein.^26^ In addition, *Plasmodium yoelii* and *Plasmodium falciparum* sporozoites utilize a specific CD81-dependent pathway to enter hepatocytes.^28^ We, thus, addressed whether CD81 is involved in *M. abscessus* (*Mab*) uptake, subsequently leading to macrophage infection. The requirement of CD81 for the internalization of *Mab* by macrophages was suggested using neutralizing monoclonal antibodies specifically directed against CD81-LEL (Figure 1A). Results show that pre-treatment of human THP-1 cells with the antibodies before *Mab* infection effectively inhibited bacilli invasion of macrophages and significantly reduced the intracellular bacterial burden. As negative controls, IgG1 isotype or monoclonal antibodies raised against tetraspanins CD82 and CD151, had no impact on *Mab* uptake by macrophages (Figure 1A). Unexpectedly, neutralization of CD81 reduced *Mab* invasion to the same extent than neutralization of CD43, mannose receptor (MR) and complement receptor 3 (CR3) (Figure 1A), known as major PRR participating in the invasion of *Mycobacterium tuberculosis*.^30^^,^^31^^,^^32^^,^^33^^,^^34^ Similar results were obtained when assaying primary human monocyte-derived macrophages (MDM) (Figure 1B). In parallel, quantification of infected macrophages revealed a marked reduction in the percentage of *Mab*-containing cells when pre-treated with anti-CD43, MR, CR3, and CD81 antibodies (Figures 1C, 1E, and S1). This was not observed in THP-1 cells either pre-incubated with the IgG1 isotype control, anti-CD82 or anti-CD151 antibodies (Figures 1C and 1E). To further assess the consequences of this invasion defect, infected macrophages were classified into three categories at 3 hours post-infection (hpi) based on their bacterial content: weakly infected (<5 bacilli/cell), moderately infected (5–10 bacilli/cell), and heavily infected (>10 bacilli/cell) (Figure 1D). The results indicate that blocking CD81, but not CD151, profoundly increased the proportion of the weakly infected category while reducing the percentage of heavily infected cells (Figures 1D and 1E). Together, this suggests that CD81 is involved in *Mab* uptake by macrophages.

### Peptide competition with CD81-LEL reduces mycobacterial entry into macrophages

**Figure 2:**
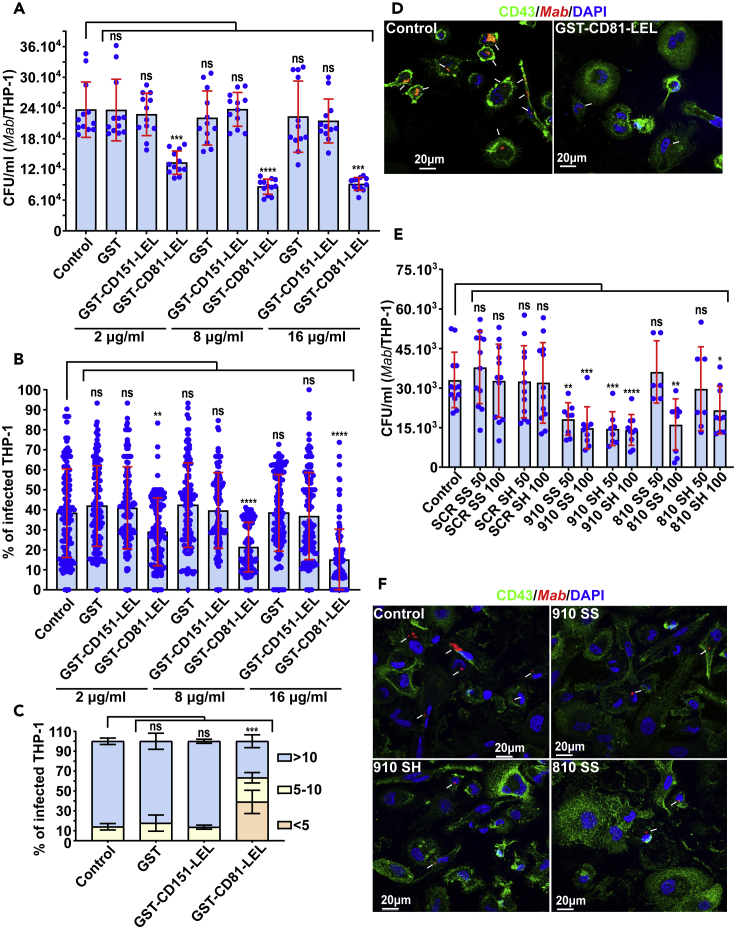
Recombinant GST-CD81-LEL or CD81-LEL derived peptides reduce *Mab* internalization by macrophages (A) Uptake of *Mab* is reduced in THP-1 macrophages pre-treated with recombinant GST-CD81-LEL at different concentrations (2, 8 and 16 μg/mL). Macrophages were lysed with 1% Triton X-100 and serial dilutions were plated before CFU counting. Data are mean values ±SD for four independent experiments (each time in triplicate) (n = 12). One-tailed Tukey’s test: ns, non-significant > 0.05, ∗∗∗p < 0.001, ∗∗∗∗p < 0.0001. (B) Pre-incubation of *Mab* with different concentrations of recombinant GST-CD81-LEL (2, 8 and 16 μg/mL) significantly reduces the bacterial uptake and percentage of infected macrophages. Quantification of the percentage of cells containing bacilli was performed as in Figure 1C. Data are mean values ±SD for six independent experiments (n = 120 fields). One-tailed Tukey’s test: ns, non-significant > 0.05, ∗∗p < 0.01, ∗∗∗∗p < 0.0001. (C) Percentage of macrophage categories after pre-treatment with recombinant GST-CD81-LEL at 16 μg/mL. The number of bacilli/macrophage drops when bacilli were pre-incubated with CD81-LEL. Values are means ± SD for three independent experiments performed each time in triplicate (n = 900 infected macrophages). One-tailed non-paired t test: ns, non-significant > 0.05, ∗∗∗p < 0.001. (D) Two immunofluorescent fields taken at 3 h post-infection using a confocal microscope (40× magnification), showing the macrophages infected with *Mab* (in red) after pre-treatment with GST-CD81-LEL. The nuclei are shown in blue, the CD43 protein associated with the plasma membrane of macrophages is in green. White arrows indicate *Mab* inside the macrophage. (E) Uptake of *Mab* is reduced in macrophages when bacilli were pre-treated with CD81-LEL-derived peptides (810 and 910) either in a linear form (reduced SH) or in a cyclic form (oxidized SS) at 50 or 100 μM. A scrambled (SCR) peptide in its reduced and oxidized form is used as negative control. To determine the number of internalized bacilli, cells were lysed with 1% Triton X-100 and lysates were plated on LB agar before CFU counting. Data are mean values ±SD for four independent experiments performed each time in triplicate (n = 12). One-tailed Tukey’s test: ns, non-significant > 0.05, ∗p < 0.1, ∗∗p < 0.01, ∗∗∗p < 0.001, ∗∗∗∗p < 0.0001. (F) Four immunofluorescent fields taken at 3 h post-infection using a confocal microscope (40× magnification), showing the macrophages infected with *Mab* (in red) after pre-treatment with CD81-LEL-derived or SCR peptides. The nuclei are shown in blue, the CD43 protein associated with the plasma membrane of macrophages is in green. White arrows indicate *Mab* inside the cells.

To assess whether CD81 acts as a macrophage receptor for the bacilli through its LEL domain, soluble GST-tagged CD81-LEL fusion protein (GST-CD81-LEL) produced in *Escherichia coli* was used in competition assays with *Mab* in THP-1 cells. CD151-LEL was also produced and included as a control. Bacteria were pre-incubated with the two GST-LEL proteins (Figure S2A), separately for 3 h at 37°C before infection. In contrast to GST-CD151-LEL, pre-incubation with GST-CD81-LEL significantly reduced both the intracellular bacterial burden (Figure 2A) and the percentage of *Mab*-infected THP-1 cells (Figures 2B, 2D, and S2B), relative to the GST control. A marked increase in the proportion of the low-infected macrophage category was also observed when bacilli were pre-incubated with GST-CD81-LEL, but not with GST-CD151-LEL or GST alone (Figures 2C and 2D). This inhibitory effect on bacterial entry was also dose-dependent, supporting both a qualitative and quantitative effect of the fusion protein-mediated competition (Figures 2A and 2B).

Next, two short peptides (810 and 910) exposed and located between the 3 cysteine residues (Cys157, Cys175 and Cys190) found in CD81-LEL (Figures S3A and S3B) were synthesized in their linear (SH) or cyclic (SS) forms and tested for their activity to influence *Mab* uptake by THP-1 cells. A scrambled peptide (SCR), consisting of the same amino acids in random order, was included as an internal control. *Mab* were pre-incubated with different concentrations of peptides, ranging from 50 μM to 100 μM. Significant reductions in bacterial uptake were observed with both the 810 and 910-derived peptides (Figures 2E, 2F, and S3C). These results indicate that both the linear and cyclic forms of peptide 910 are equally effective and act at lower concentrations than peptide 810 to block mycobacterial internalization. As anticipated, bacteria pre-treated with the SCR peptide showed no reduction in their ability to invade the macrophages (Figures 2E and S3C). Collectively, these data establish the importance of the LEL short loops formed between the disulfide bridges in the interaction with the bacterial ligand(s) and required for the internalization process.

### Deletion of the CD81-LEL reduces bacterial internalization in lung epithelial cells

**Figure 3:**
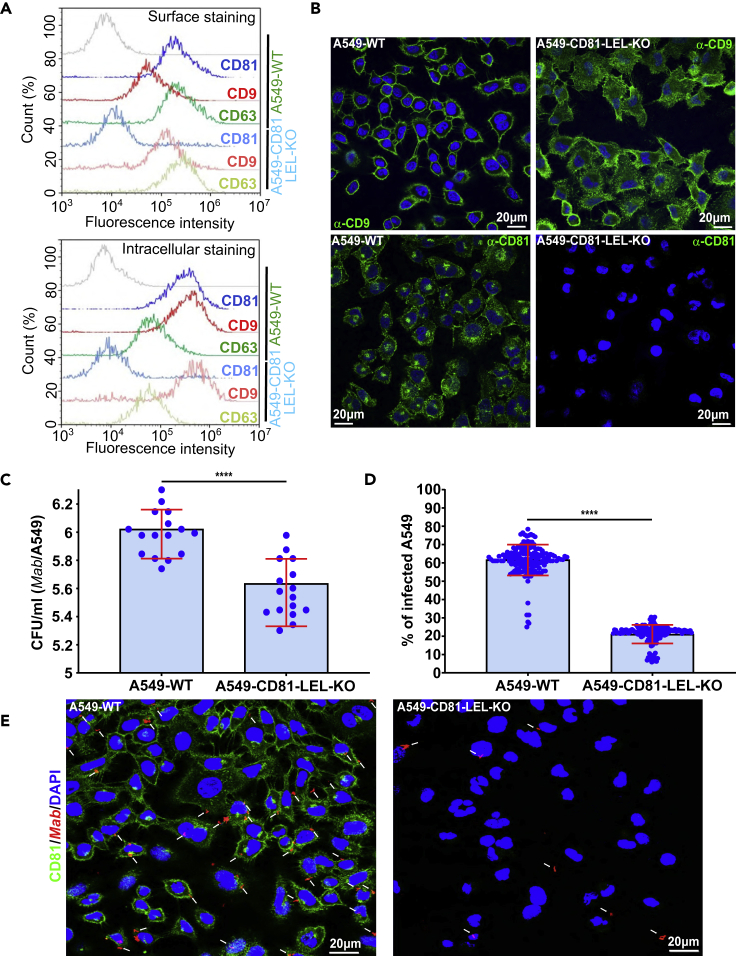
Deletion of CD81-LEL in lung epithelial cells reduces internalization of bacilli and intracellular bacterial loads (A) A549-WT or A549-CD81-LEL-KO cells were harvested and fixed in 2% paraformaldehyde before washing and incubated with indicated primary antibodies, followed by staining with Alexa Fluor 647-coupled secondary antibodies in non-permeabilizing (surface staining) or permeabilizing (intracellular staining) buffer. Cells were then washed and processed through a Novocyte ACEA cytometer. Surface staining (upper inset) and intracellular staining (lower inset) are represented as histogram overlays for each tetraspanin expression in both cell types (CD81 in blue; CD9 in red; CD63 in green). The staining control was done with incubation of secondary antibodies alone (gray curves). (B) Localization of tetraspanins CD9 and CD81 (green staining) in A549_WT cells and in A549-CD81-LEL-KO cells. Because these antibodies are directed against the LEL, no CD81 signal is detected in A549-CD81-LEL-KO cells. (C) Impact of CD81-LEL disruption in A549 cells on the internalization of *Mab*. CFUs were determined at 3 h post-infection, as described previously. Data are the means ± SD values for four independent experiments performed in quadruplicate (n = 16). One-tailed Tukey’s test: ns, non-significant > 0.05; ∗∗∗∗p < 0.0001. (D) Deletion of CD81-LEL significantly diminishes the uptake of *Mab* and the percentage of infected A549 cells. Quantification of the percentage of host cells containing bacilli was performed as in Figure 1C. Data are mean values ±SD for eight independent experiments (n = 160 fields). One-tailed Tukey’s test: ns, non-significant > 0.05, ∗∗∗∗p < 0.0001. (E) Immunofluorescence confocal microscopy fields showing A549 WT, or A549-CD81-LEL-KO cells infected with red-fluorescent mycobacteria (white arrows). The cell surface is stained using anti-CD81 antibodies (green) whereas the nuclei are stained with DAPI (blue).

To confirm the role of CD81-LEL in *Mab* internalization, CRISPR/Cas9-mediated genome editing was applied to A549 lung epithelial cells, yielding to A549-CD81-LEL-KO cells. A guide RNA targeting the exon 4 of *Cd81* gene just upstream of the LEL coding DNA region was designed. Guide RNA (sgRNA) sequence targeting *Cd81* was designed and cloned into the LentiGuide-Puro (Figure S4A).^35^ Viral particles generated in HEK293T cells were used to knock-out the LEL of *Cd81* genes specifically in A549 cells by lentiviral transduction and single-cell cloning (Figure S4A). A549-CD81-LEL-KO cells were further sorted by flow cytometry and isolated into a single cell by limiting dilution. One single clone was expanded and selected for further analysis. Specific introduction of indels at the sgRNA cut side in A549 cells was validated by PCR and complete sequencing of *Cd81* open reading frame (Figure S4B). Cloning of the PCR products obtained from the cDNA prepared from clone A549-CD81-LEL-KO cells into the TOPO vector, followed by sequencing, revealed the existence of two different deletions, presumably representing two different genomic repairs occurring at the 2 *Cd81* alleles (Figure S4B). The first one exhibited a 5-bp deletion in the translated region of *Cd81* gene, which would generate a chimeric and short protein lacking LEL (Figures S4B and S4C), whereas the second one revealed a 20-bp deletion that also generated a chimeric and truncated protein lacking the LEL (Figures S4B, and S4C). Flow cytometry and immunofluorescence analyses using anti-CD81-LEL antibodies confirmed the absence of CD81-LEL expression at the cell surface as well as intracellularly, when compared with the unedited controls (Figures 3A and 3B). No defect in the expression levels of tetraspanins CD9 and CD63 were observed in A549-CD81-LEL-KO as compared to A549-WT cells (Figures 3A and 3B). Expression of the truncated CD81 protein was confirmed by sequencing analysis. Overall, this underscores the successful and specific deletion of CD81-LEL in the A549-CD81-LEL-KO cells. Importantly, infection of A549-CD81-LEL-KO cells showed a significant decrease in the number of internalized *Mab* as compared to WT A549 cells (Figures 3C and 3E). Quantitative analysis revealed also a marked reduction in the percentage of *Mab*-containing cells in the absence of CD81-LEL (Figures 3D and 3E). These results are fully consistent with those from the pre-treatment experiments using either neutralizing anti-CD81 antibodies or recombinant GST-LEL fusion proteins (Figures 1 and 2). They demonstrate that surface-associated CD81 plays a crucial role in internalization of *Mab* by macrophages and lung epithelial cells in a CD81-LEL-dependent manner.

### Identification of CD81 ligands in *M. abscessus*

**Figure 4:**
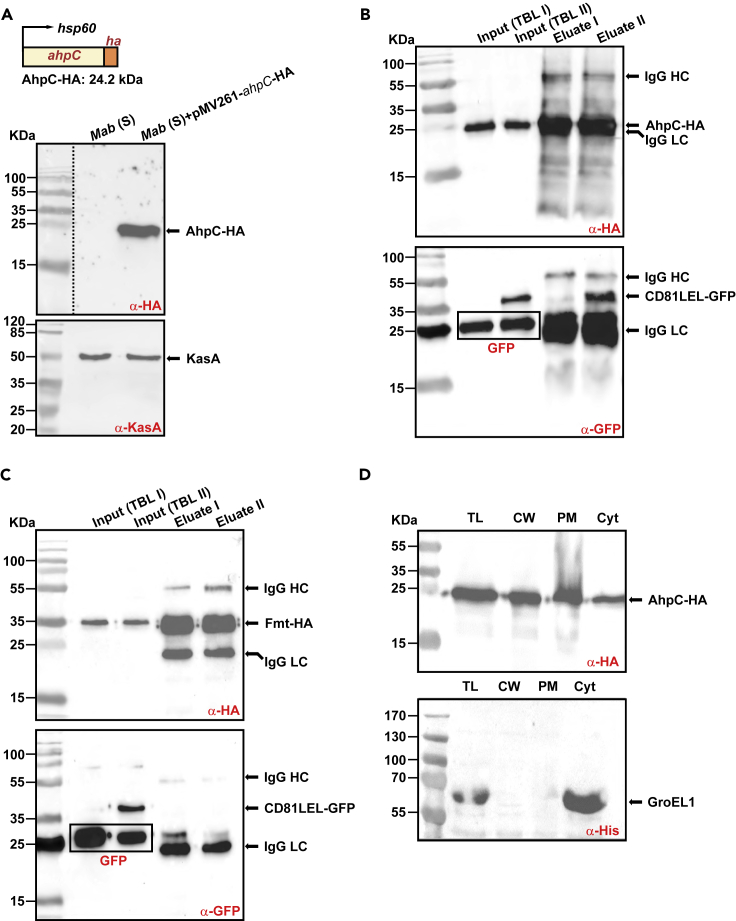
AhpC interacts specifically with CD81-LEL (A) Overexpression of AhpC-HA in *Mab*. The *ahpC* (*MAB_4408c*) gene was fused to HA and expressed under the control of *hsp60* promoter (see also Table S1). Upper panel: Western blotting of AhpC overexpression in *Mab* using anti-HA antibodies. Lower panel: The KasA protein (probed with anti-KasA antibodies) was used as a loading control. (B) Interaction of CD81-LEL-GFP with *Mab* AhpC. Co-immunoprecipitation of the complex CD81-LEL-GFP/AhpC-HA was carried out with anti-HA antibodies from *Mab* extracts. Immunoprecipitates from control (*Mab* co-expressing GFP and AhpC-HA; lane 3) or from *Mab* co-producing CD81-LEL-GFP and AhpC-HA (lane 4) were eluted and immunoblotted using either anti-HA (upper blot) or anti-GFP (lower blot) antibodies. Lanes 1 and 2 allow to detect AhpC-HA or GFP or CD81-LEL-GFP proteins in the TBL I and TBL II bacterial soluble extracts before proceeding to the immunoprecipitation experiments using beads coated with anti-HA antibodies. LC: immunoglobulin light chain; HC: immunoglobulin heavy chain. (C) The Fatty acid methyltransferase (Fmt) from *Mab* does not interact with CD81-LEL-GFP. The experiments were conducted as indicated in (B) by replacing AhpC-HA by Fmt-HA in the assay. (D) Immunoblotting showing the localization of AhpC in different fractions: total lysate (TL), cell wall (CW), plasma membrane (PM), and cytosol (Cyt). The cytosolic marker GroEL1 was included as a control.

The mycobacterial protein partners possibly interacting with CD81 are not known. To uncover putative ligands in *Mab*, a GFP-Trap pull-down approach was developed using anti-GFP nanobodies on lysates derived from mycobacteria expressing either GFP or CD81-LEL-GFP (Figures S5A and S5B) followed by mass spectrometry analyses to identify the immuno-precipitated proteins (Figure S5C). After several washes, one-fifth of each eluate was subjected to SDS-PAGE and silver staining (Figure S5D). Although a few proteins were bound to GFP- or to CD81-LEL-GFP-coated beads, the samples showed very different profiles (Figure S5D). Mass spectrometry analysis on selected proteins which were not present in the control eluate yielded at least five unique peptides (Figure S5E). Among these, a heat shock protein (Hsp16.7), an antioxidant enzyme (AhpC), and two hypothetical proteins (MAB_3498c and MAB_1957) (Figure S5E). This suggests that these potential uncharacterized protein partners may participate in the interaction between *Mab* and CD81. We then focused on AhpC (encoded by *MAB_4408c*), detected with the most unique peptides by mass spectrometry (11 versus 5 for the other candidates) and whose orthologue in *M. tuberculosis* is an alkyl hydroperoxide reductase playing a specific role in antioxidant defenses.^36^ Subsequent validation was based on a reverse HA-Trap pull-down approach. First, a *Mab* strain expressing AhpC-HA under the control of the *hsp60* promoter was generated and expression was checked by western blotting using anti-HA antibodies (Figure 4A). Second, bacterial lysates from the strain expressing AhpC-HA were mixed with lysates from *Mab* strains expressing either GFP or CD81-LEL-GFP to form two total bacterial lysates, designated TBL-I and TBL-II, respectively, and incubated with beads coupled to anti-HA antibodies (Figure S6). HA-Trap pull-down and subsequent western blotting using anti-GFP or anti-HA antibodies revealed the presence of CD81-LEL-GFP, GFP or AhpC-HA in the eluted fractions (Figure 4B). Importantly, a single band with the appropriate size of CD81-LEL-GFP was specifically pulled down with AhpC-HA but not with GFP alone or with Fmt-HA (a fatty acid methyltransferase from *Mab*), both used as negative controls (Figures 4B and 4C). This confirms the specific interaction between AhpC and CD81-LEL-GFP.

To determine whether AhpC is located in the cell wall fraction where it could exert its role as a surface adhesin to interact with CD81, sequential centrifugation/ultracentrifugation steps of total lysates from the AhpC-HA-overexpressing strain led to several fractions enriched for cell wall (CW), plasma membrane (PM) or cytosol (Cyt) proteins. SDS-PAGE followed by immunoblotting using anti-HA antibodies revealed a strong signal at 25 kDa in all fractions, in agreement with the predicted molecular weight of AhpC-HA (Figure 4D). To exclude the possibility of contaminating cytosolic proteins in the CW and PM fractions, membranes were probed with anti-His antibodies to detect the cytosolic GroEL1 protein (Figure 4D). These results support the view that AhpC is a cell wall-associated protein, thus ideally exposed to interact with CD81 (Figure 4D).

### AhpC promotes *Mab* uptake by macrophages via CD81

**Figure 5:**
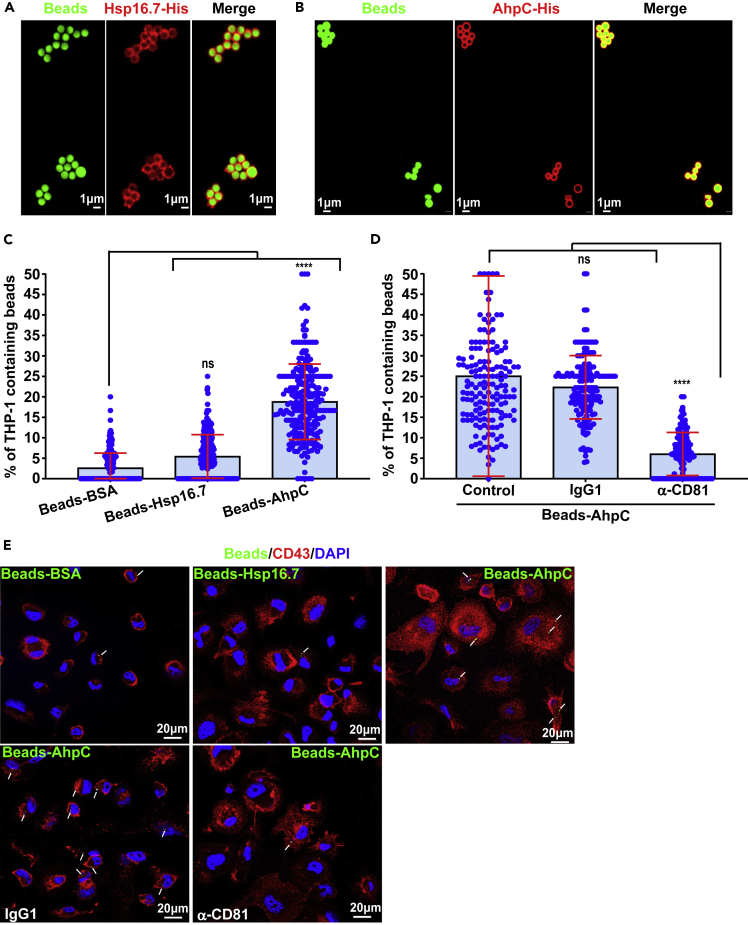
AhpC interacts directly with CD81 and promotes internalization of fluorescein-tagged latex beads (A and B) Protein coating of fluorescein-tagged latex beads with recombinant *Mab* Hsp16.7-His or AhpC-His. Labeling of Hsp16.7-His or AhpC-His proteins (red) at the surface of fluorescein tagged latex beads (green) was detected using mouse anti-His antibodies and Alexa Fluor 594 conjugated goat anti-mouse secondary antibodies. (C) Ingestion of beads by THP-1 macrophages for 4 h is optimal when coated with AhpC. Data are mean values ±SD for three independent experiments (each time in octuplicate) (n = 240 fields). One-tailed Tukey’s test: ns, non-significant > 0.05, ∗∗∗∗p < 0.0001. (D) Blocking CD81 with neutralizing antibodies drastically reduces the uptake of AhpC-coated beads. Data are mean values ±SD for four independent experiments (each time in quadruplicate) (n = 160 fields). One-tailed Tukey’s test: ns, non-significant > 0.05, ∗∗∗∗p < 0.0001. (E) Representative fluorescent images showing either the efficiency of internalization and impact of neutralizing anti-CD81 antibodies on the internalization of AhpC-coupled beads by macrophages. CD43 (red), DAPI (blue) and fluorescent beads (green). White arrows indicate fluorescent beads inside macrophages.

*Mab* AhpC and Hsp16.7, both identified by mass spectrometry (Figure S5E), were produced and purified as recombinant AhpC-6×His and Hsp16.7-6×His proteins from *E. coli* (Figures S7A and S7B). Fluorescent latex beads (1 μm diameter) were coated with either AhpC-6×His, Hsp16.7-6×His or BSA. The efficacy of coating was confirmed by SDS-PAGE/western blotting (Figures S7C and S7D) and by immunofluorescence using anti-His or anti-BSA antibodies (Figures 5A, 5B, and S7E). The effect of protein coating on beads uptake was next assessed by incubating THP-1 macrophages with the different fluorescein-labeled latex beads. Quantification of the percentage of bead-containing cells clearly demonstrated that macrophages internalized significantly more AhpC-coated beads (5- to 10-fold) than Hsp16.7-, or BSA-coated beads (Figures 5C and 5E). Strikingly, the uptake of AhpC-beads was inhibited by pre-treating macrophages with anti-CD81 antibodies but not in the presence of the IgG1 control isotype (Figures 5D and 5E). This validates the physical interaction between AhpC and CD81 to favor the internalization of the beads by macrophages. High-resolution confocal imaging also highlighted the intracellular localization of the counted beads (Figure 5E). Collectively, these findings show that mycobacterial AhpC acts as a ligand for the host CD81 and that internalization of whole bacteria by host cells is dependent on the AhpC/CD81 pair.

### Overexpression of *ahpC* enhances AhpC-mediated cell entry

**Figure 6:**
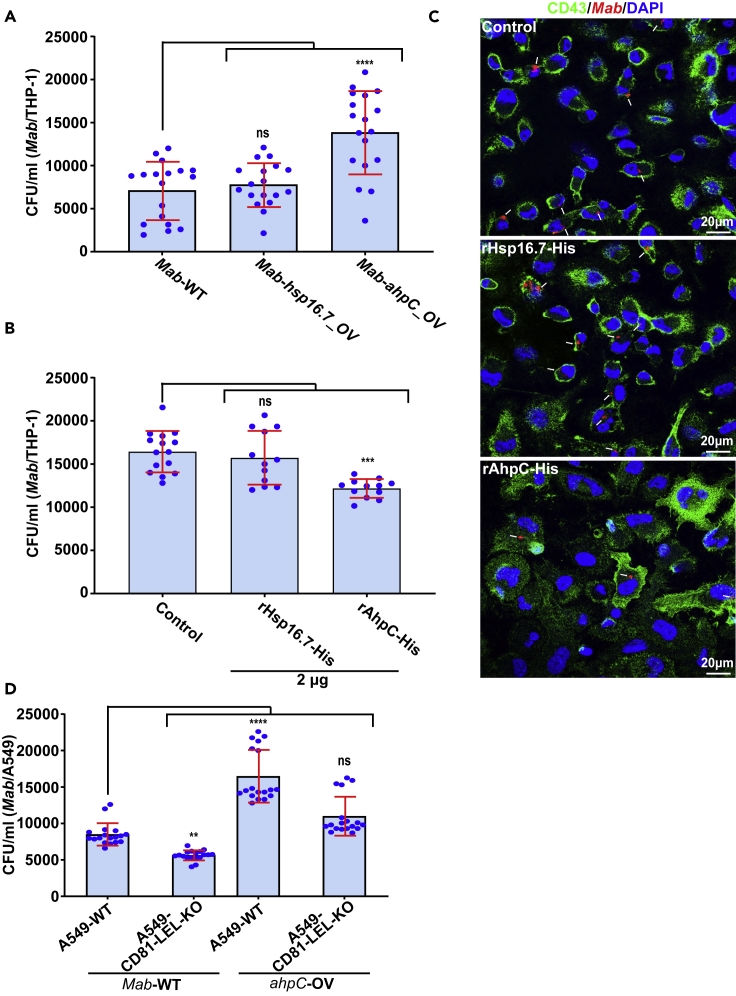
Overexpression of AhpC enhances colonization of macrophages by *Mab* (A) Effect of overexpression of AhpC or Hsp16.7 on the ability of *Mab* to invade THP-1 macrophages (see also Table S1). CFU were calculated as reported above. Data are mean values ±SD for three independent experiments, each time in sextuplicate (n = 18). One-tailed Tukey’s test: ns, non-significant > 0.05, ∗∗∗∗p < 0.0001. (B) Pre-treatment of THP-1 cells with 2 μg of soluble recombinant Hsp16.7-His or AhpC-His on the ability of *Mab* to colonize THP-1 human cells. CFU were determined as described above. Data are mean values ±SD for three independent experiments, each time in triplicate (n = 12). One-tailed Tukey’s test: ns, non-significant > 0.05, ∗∗∗p < 0.001. (C) Three immunofluorescent fields taken at 3 hpi using a confocal microscope (40× magnification), showing the macrophages infected with *Mab* (in red) after pre-treatment of THP-1 cells with Hsp16.7-His or AhpC-His recombinant proteins. Nuclei are in blue and the cell surface is in green. White arrows indicate mycobacteria inside macrophages. (D) Overexpression of AhpC enhances entry of bacilli into A549-WT in a CD81-LEL-dependent manner. CFU were assessed at 3 hpi as outlined earlier. Data are mean values ±SD for three independent experiments, each time in sextuplicate (n = 18). One-tailed Tukey’s test: ns, non-significant > 0.05, ∗∗p < 0.01, ∗∗∗∗p < 0.0001.

Whether overproduction of AhpC interferes with bacterial invasion was tested by overexpressing *ahpC* (as well as *hsp16.7*) in *Mab*. For this purpose, the episomal pMV261 carrying either *hsp16.7* or *ahpC* fused to an HA-tag under the control of the *hsp60* promoter was introduced into *Mab* and the invasion rates of *Mab*_WT, *Mab*_*hsp16.7*_OV or *Mab*_*ahpC*_OV were compared. As shown in Figure 6A, the internalization of *Mab-S-ahpC_*OV was 2-fold higher than internalization of *Mab_*WT or *Mab*_*hsp16.7*_OV, suggesting that AhpC is acting as a mycobacterial adhesin. To further assess the impact of AhpC on infection, THP-1 cells were pre-incubated with recombinant AhpC-His before macrophage infection with whole bacteria. Pre-treatment with 2 μg AhpC-His partially blocked internalization of the *Mab*_WT and reduced infectivity by 30% as compared to untreated cells or to cells pre-incubated with recombinant Hsp16.7-His (Figures 6B and 6C).

To validate the role of CD81 in interacting with AhpC on whole bacteria, we investigated the ability of *Mab*_WT and *Mab*_*ahpC*_OV strains to invade A549-WT and A549-CD81-LEL-KO cells. CFU determination indicates that although overproduction of AhpC in *ahpC-*OV enhances *Mab* internalization by A549 cells (confirming the results obtained in macrophages), the uptake of *Mab*_*ahpC*-OV is significantly reduced in A549-CD81-LEL-KO cells as compared to the uptake of *Mab*_WT in these cells (Figure 6D). This emphasizes the requirement of CD81 for optimal internalization of the AhpC-overproducing strain. Overall, these results indicate that whereas overproduction of AhpC facilitates *Mab* internalization, pre-incubation of cells with soluble AhpC inhibits the internalization process, thus indicating that AhpC behaves like a canonical adhesin.

### Conditional deletion of *ahpC* reduces invasion of *Mab*

**Figure 7:**
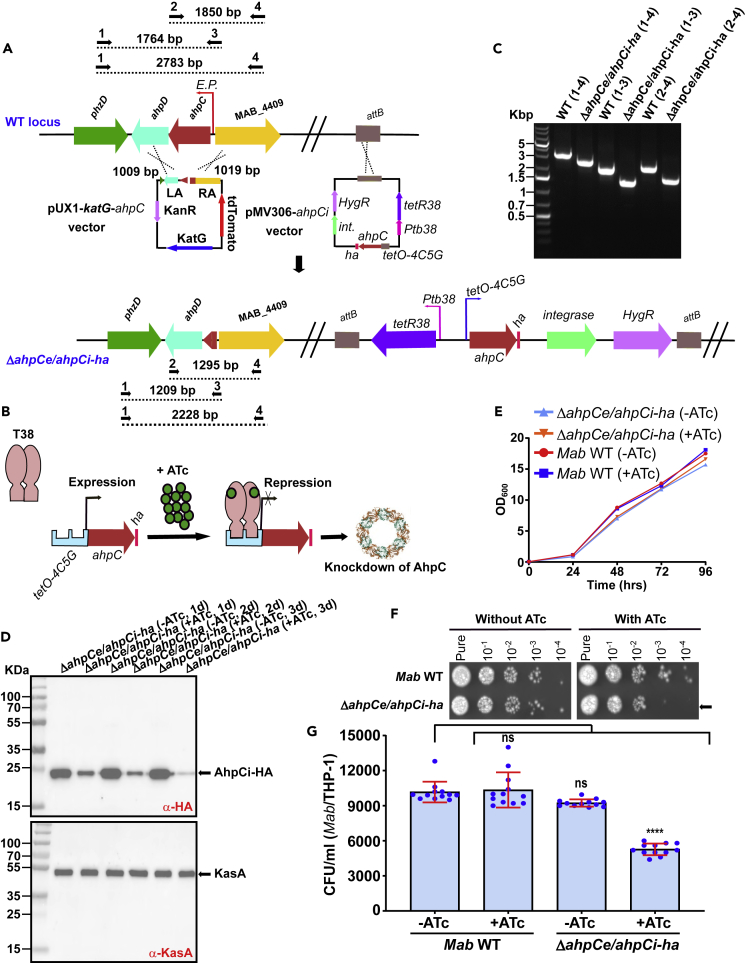
Internalization of *Mab* by macrophages requires the AhpC adhesin (A) Schematic illustration of the *Mab ahpC* conditional knock-out strategy (see also Table S1). *Mab* was transformed with the regulatory, *ha*-tagged *ahpCi* plasmid (pMV306-*ahpCi*) to generate a merodiploid bacterial strain. The pMV306-*ahpCi* vector was integrated into the *attB* site by single homologous recombination. The pMV306-*ahpCi* contains the integrase gene (*int*), the hygromycin resistance cassette (*HygR*), the *tetR38* repressor under the control of the *Ptb38* promoter, and the C-terminal *ha*-tagged *ahpC* gene under the control of the inducible *tetO-4C5G* promoter. The single merodiploid clone was then transformed with pUX1-*katG*-*ahpC* to remove the endogenous *ahpC* gene (*ahpCe*) by double homologous recombination. The *ahpC* gene is sandwiched between the *ahpD* and *MAB_4409*. The DNA sequences of the left and right arms of *ahpC* were amplified by PCR and subcloned into pUX1-*katG*. The resulting suicide plasmid was used to transform the strain expressing an additional HA-tagged copy of *ahpC* gene. Dotted lines represent the size (indicated above each line) of the expected PCR products in *Mab* WT, and Δ*ahpCe/ahpCi-ha*. Black arrows represent the primers used for PCR analysis. (B) Tetracycline-mediated regulation of the *ahpC* gene using the tet-OFF configuration in *Mab*. The *tetR38* gene encoding the T38 repressor is a reverse TetR that recognizes *tetO-4C5G*. Addition of ATc results in binding of T38 to ATc and its recruitment to *tetO-4C5G* represses the transcription of the HA-tagged *ahpCi* gene. T38, Tet repressor; ATc, anhydrotetracycline; *tetO*, Tet operator. (C) PCR analysis confirming the deletion of *ahpC* in *ΔahpCe/ahpCi-ha*. Genomic DNA from *Mab* was used to amplify the intact *ahpC* locus. Amplicon was subjected to sequencing to confirm the proper deletion of *ahpC*. (D) Time-dependent depletion of the inducible AhpCi-HA upon addition of ATc to the medium is shown after 24 h, 48 h, and 72 h in *ΔahpCe/ahpCi-ha* (upper panel). KasA was used as a loading control (lower panel). (E) Parental *Mab* and *ΔahpCe/ahpCi-ha* strains growth curves in 7H9 media supplemented or not with ATc at 37°C. The data are from one of two independent experiments. (F) Bacteria were grown to exponential phase and 1.5 μL of 10-fold serial dilutions were spotted onto LB agar medium supplemented or not with 1 μg/ml ATc. Pictures were taken after 5 days of incubation at 37°C. Arrows indicate the difference of growth in presence or absence of ATc. (G) Comparison of the invasion capacity of the parental and *ΔahpCe/ahpCi-ha* strains pre-exposed to 1 μg/mL ATc for 72 h and subsequently used to infect THP-1 cells. CFUs were determined at 3 hpi as reported earlier. The results represent the mean values ±SD of three independent experiments (each conducted in triplicate) (n = 9). One-tailed Tukey’s test: ns, non-significant > 0.05, ∗∗∗p < 0.001.

Host cell invasion by mycobacteria is dependent on interactions between bacterial adhesins with moonlighting activity and host cell receptors.^37^ This may explain why some adhesins are essential for *in vitro* growth. Despite numerous attempts, we were unable to generate an *ahpC* deletion mutant using the unmarked deletion strategy, previously validated for the deletion of non-essential genes in *Mab*.^8^^,^^38^^,^^39^ Consequently, to investigate the contribution of AhpC in bacterial uptake by macrophages, we took advantage of the tetracycline repressor system that selectively and ectopically controls gene expression in mycobacteria.^40^^,^^41^ To generate a conditional *ahpC* mutant, we introduced the *ahpC* minigene with a C-terminally positioned HA epitope tag into *Mab* (Figure 7A). This minigene was placed under the control of the tetracycline regulatory promoter *tetO-4C5G* and integrated into the bacterial *attB* locus (designated *ahpCi-ha*) following selection on hygromycin (Figures 7A and 7B). The resulting *ahpCe/ahpCi-ha* strain was used to disrupt the endogenous copy of *ahpC* (designated *ahpCe*) by double homologous recombination using the pUX1-*katG*-*ahpC* vector containing two flanking regions (1009 bp and 1019 bp) of the *ahpC* gene (Figure 7A). The resulting clones were analyzed by PCR, confirming the proper genotype of the mutant (Figures 7A and 7C). In addition, western blotting was performed to select for clones in which a significant reduction in AhpC-HA expression was observed following treatment with anhydrotetracycline (ATc) (Figure 7D). The knockdown of AhpCi-HA protein in presence of ATc was monitored by western blotting at days 1, 2, and 3 using an anti-HA antibody (Figure 7D). Expression of AhpCi-HA was strongly downregulated in the presence of 1 μg/mL ATc for 72 h (Figure 7D). Based on these observations, all subsequent invasion experiments were conducted using the Δ*ahpCe/ahpCi-ha* conditional mutant pre-treated with 1 μg/mL ATc for 72 h.

Monitoring bacterial growth failed to show any *in vitro* growth defect on depletion in AhpCi-HA in planktonic cultures during the first 96 h of incubation in the presence of ATc (Figure 7E). However, a more fine-grained growth analysis performed by taking 1.5 μL of the Δ*ahpCe/ahpCi-ha* culture treated in the presence of 1 μg/mL of ATc showed a slight growth defect on agar plates as evidenced by a decreased number of colonies at the highest dilutions (Figure 7F). As expected, the parental *Mab* strain grew similarly in the presence or absence of ATc (Figure 7F) whereas in the absence of ATc, the Δ*ahpCe/ahpCi-ha* mutant grew similarly to the WT strain. This suggests that AhpC is likely to play a minor role for *in vitro* growth. Next, bacteria pre-treated for 72 h with or without ATc were used to infect THP-1 macrophages. At 3 h post-infection, only Δ*ahpCe/ahpCi-ha* pre-exposed to ATc showed a reduced capacity to invade the cells (Figure 7G). This unambiguously establishes that depletion of AhpC reduces mycobacterial invasion.

## Discussion

Mycobacteria express various PAMP, which directly interact with their cognate PRR, including TLR, CLR, and other receptors.^42^ The interplay between macrophages and mycobacteria is complex and is not fully understood. Receptors used by several important human pathogens include tetraspanins, which form adhesion platforms and facilitate internalization of these microorganisms.^22^^,^^43^^,^^44^^,^^45^ Tetraspanins are involved in the pathogenesis of infections caused by viruses, parasites, protozoa and bacteria at various levels.^46^ Evidence is accumulating that these membrane proteins are involved in the adhesion and uptake of some bacteria, such as *Chlamydia*, *Listeria* or *Neisseria*^22^ whereas their contribution in the recognition of mycobacteria remains unknown. Herein, we demonstrate that tetraspanins participate in the interplay between *Mab* and host cells, such as macrophages and lung epithelial cells. We showed that tetraspanin CD81 is an abundant protein expressed on the surface of phagocytic and epithelial cells, whereas CD151 is present at the surface of host cells at lower levels.^47^^,^^48^ We provide compelling evidence that CD81-LEL is crucial for mycobacterial internalization. Interfering with CD81-LEL using neutralizing antibodies caused a significant reduction in *Mab* uptake by macrophages (THP-1 and primary human macrophages) and pneumocytes. That no inhibition was observed with the LEL of CD151 attests for the specificity of the interaction. Moreover, CD81-LEL-derived peptides considerably reduced the internalization of *Mab* by macrophages. It is tempting to speculate that these peptides can act similarly to the recombinant CD81-LEL, by preventing the interaction between the bacilli and the macrophage. This is of particular interest given that anti-adherence therapies represent a relatively new area of research to combat infectious diseases.^49^ Indeed, most commercially available antimicrobials inhibit the growth of the pathogens but this selective pressure can rapidly lead to the emergence of drug-resistant strains. Unlike traditional drugs, anti-adhesion peptides that target host and bacterial components show a reduced probability of developing resistance.^22^^,^^50^^,^^51^ Small synthetic peptides based on tetraspanin CD9 prevent adhesion of *Staphylococcus aureus* to human keratinocytes and in a model of human skin.^51^ This property, together with their high specificity and low toxicity, make tetraspanin-derived peptides attractive candidates for future antibacterial drug developments.

The requirement of CD81 for optimal internalization of *Mab* was confirmed by knocking-out CD81-LEL using CRISPR/Cas9 strategy in A549 pneumocytes. Other reports indicate that tetraspanins indirectly contribute to the process of bacterial internalization, either by acting as a co-receptor or by recruiting the receptor to the cell surface via the assembly of tetraspanin-enriched microdomains (TEM) to facilitate bacterial binding.^22^ It is very likely that TEM are involved in the adhesion of *Salmonella typhimurium* to human monocyte-derived macrophage and that this may represent a common mechanism that a variety of bacteria exploit as a mean of attachment before invasion.^52^ The inhibitory effect seen on internalization of bacilli when interfering with the CD81-LEL function may be due to the disruption of interactions between cell surface tetraspanins and adhesion receptors, resulting in the disorganization of the adhesion platforms required for optimal bacterial attachment. However, this hypothesis can be dismissed in our study because we have identified, for the first-time, a bacterial ligand that interacts directly with the LEL of a tetraspanin in a context where TEM are absent.

Although mass spectrometry analysis identified several putative ligands of CD81-LEL, we primarily focused here on the alkyl hydroperoxide reductase C (AhpC). *M. tuberculosis* AhpC belongs to the peroxiredoxin family, acting as a general antioxidant enzyme by reducing alkyl hydroperoxides more effectively than H_2_O_2_ and protecting bacilli from oxidative stress.^53^ In *Mab*, AhpC was found to be associated with the cell wall and the cytosolic fractions. Reactive oxygen species and reactive nitrogen intermediates released by phagocytic cells play an important role in the defense against intracellular mycobacteria. It is very likely that AhpC contributes to thwarting the generation of oxidative stress and damage, and consequently protects the bacilli from the oxidative burst, although this needs to be investigated in future studies. In addition, the localization of AhpC on the bacterial surface conceivably facilitates adherence of the bacilli to host cells, where it may act as an adhesin interacting with CD81. Alternately, because of the co-expression of multiple adhesins, blocking one adhesin at a time may not be sufficient to affect the binding of whole bacilli to macrophages. The specificity of the physical interaction between AhpC and CD81 was demonstrated using a combination of *in vitro* based approaches: (1) Macrophage uptake of AhpC-coated latex beads was higher than that uptake of BSA- or Hsp16.7-coated beads; (2) The uptake of AhpC-coated beads was reduced when macrophages were pre-treated with anti-CD81-LEL antibodies. Thus, these *in vitro* and *in cellulo* observations are in good agreement with each other. Future studies should be dedicated to understand how *Mab* regulates expression of AhpC to drive the interaction with CD81 during infection. In addition, whether the other CD81-LEL adhesin candidates identified in this study (MAB_3498c and MAB_1957) play a role in the early recognition of *Mab* by CD81 will deserve further attention.

Tetraspanins are versatile proteins, involved in many important cellular functions. How these cell-surface proteins generate signals that are translated into intracellular processes remains unclear. Many tetraspanin members have been shown to interact with one another, forming protein complexes that are associated with lipid rafts.^54^ These complexes can also recruit integrins as well as other receptors or proteins, which may then transduce signals triggering intracellular events.^55^^,^^56^ The aggregating property of the tetraspanins suggests that they may function as an organizer of cell-surface proteins.^57^^,^^58^ In the case of CD81, it remains to be determined whether it can activate directly or indirectly a signal transduction after its interaction with AhpC. Other tetraspanins are widely distributed on the cell surface, and their association with other cell-surface proteins can result in a wide range of cellular responses. As a result, this process is inherently complex and difficult to analyze. It is unclear at this stage whether the binding of AhpC to CD81 triggers intracellular signaling, and consequently causes biological events such as cytoskeletal changes, membrane ruffling, or release of inflammatory mediators.

Despite the clear involvement of CD81 in *Mab* uptake, inactivation of CD81-LEL does not abolish *Mab* internalization in THP-1 and A549 cells. Thus, it is very likely that CD81 plays a redundant role with other receptors. Nevertheless, that CD81 plays a key role in the entry of *Mab* is of particular interest, taking into account that CD81 has been reported to be required for the infectivity of hepatitis C virus (HCV), HIV, Listeria and Plasmodium species in hepatocytes and in other cell types.^26^^,^^28^^,^^47^^,^^59^ Moreover, cholesterol is critical for replication, secretion, and entry of HCV into target cells,^60^ and contributes to the organization of CD81-enriched microdomains required for the infectivity of Plasmodium,^61^ and it is critical for the entry of Listeria into target cells by both the InlA and InlB (internalin) entry pathways.^62^ Future studies should address whether cholesterol plays a role in the distribution of CD81 at sites of *Mab* entry and determine which events are downstream of CD81 signaling.

In conclusion, this work demonstrates that *Mab* bacilli depend partially on a specific CD81-dependent pathway to infect macrophages and pneumocytes, further substantiating the complexity of the mycobacterial interactions with host cells. It also adds a so far uncharacterized receptor/ligand pair to the growing list of actors involved in the molecular interactions between mycobacteria and macrophages. As such, this study constitutes a major step toward the elucidation of the early events leading to mycobacterial infection. Because AhpC is conserved in many mycobacterial species (∼83% of similarity), including *M. tuberculosis*, it is therefore tempting to speculate that the CD81/AhpC interaction participates also in the uptake of other pathogenic mycobacteria by phagocytic cells, which will be assessed in other studies. Throughout this study, experiments were carried out using the smooth morphotype, reported to be less virulent than the rough morphotype.^8^ Of interest, the *ahpC* gene is approximately two-fold more expressed in the rough variant as compared to the smooth variant.^63^ Therefore, further studies should focus on generating an *ahpC* mutant in the rough background and analyze the impact on uptake by macrophages and the consequences on pathogenicity. Furthermore, because CD81 represents a multi-pathogen receptor (for HCV, HIV, Plasmodium, *Mab*), targeting this receptor deserves thorough attention, as a large part of the human population is exposed to these infections.

### Limitations of the study

Although this study provides meaningful information on the so far unexpected role of surface-associated CD81 during *Mab* infection, there are some limitations that should be addressed in future studies. For instance, experiments to identify the structural domains involved in the AhpC-CD81-LEL interaction would provide a deeper characterization of this important mechanism of bacterial uptake. Furthermore, because all studies were conducted using *ex vivo* infected cells, additional work on role of CD81 in the early and late phases of *Mab* infection should be carried out using mouse models of infection. Because the present work focused essentially on *Mab*, it would be relevant to explore whether other pathogenic mycobacteria, including *M. tuberculosis,* are also dependent on CD81 during the early interaction and infection with macrophages.

## STAR★Methods

### Key resources table

**Table:** 

REAGENT or RESOURCE	SOURCE	IDENTIFIER
Bacterial Strains		

*Mab sensu stricto*, strain CIP104536^T^, smooth	Laboratoire de Référence des Mycobactéries (IP, France)	ATCC19977^T^
Δ*ahpCe/ahpCi-ha*: Unmarked deletion of *ahpC* in *Mab* S expressing an ATc-regulatory copy of *ahpC*	This study	N/A
*E. coli* XL1-Blue	Stratagene	Cat#200249

**Oligonucleotides**		

Primers are listed in Table S1 Merck N/A		

Critical Commercial Assays		

Hygromycin B	Sigma-Aldrich	Cat#H3274
Kanamycin	Euromedex	Cat#EU0420-B
Ampicillin	Euromedex	Cat#EU0400-E
Difco Middlebrook 7H9 Broth	Thermo Fisher Scientific	Cat#DF0713-17-9
Difco Middlebrook 7H10 Broth	Thermo Fisher Scientific	Cat#DF0627-17-4
Middlebrook OADC Growth Supplement	Sigma-Aldrich	Cat#M0678
Tween-80	Sigma-Aldrich	Cat#P1754
Q5 DNA Polymerase	New England Biolabs	Cat#M0491L
T4 DNA Ligase	New England Biolabs	Cat#M0202S
NucleoSpin Plasmid Kit	Machery-Nagel	Cat#740588.50
NucleoSpin Gel and PCR Clean-up	Machery-Nagel	Cat#740609.50
GenElute HP Plasmid Midiprep Kit	Sigma-Aldrich	Cat#NA0200-1KT
Pierce Silver Stain Kit	Life-Technologies	Cat#24612
DAPI Solution	Becton Dickinson	Cat#564907
In-Fusion	TAKARA	Cat#638947
Anhydrotetracycline	Merck	Cat#37919-100MG-R
Isoniazid	Merck	Cat#I3377-250G
BCA assay	Thermo Fisher Scientific	Cat#23227
BSA	New England Biolabs	Cat#B9000S
SuperSignal West Femto Maximum Sens	Life-Technologies	Cat#34095
Pierce BCA Protein Assay Kit	Thermo Scientific	Cat#23227

Recombinant DNA		

pTEC27	Takaki K et al.^64^	Lalita Ramakrishnan, Cat#30182
pUX1-*katG*	Daher W et al.^8^	N/A
pGEX-2T	Silvie O et al.^28^	N/A
pET30	Sigma-Aldrich	Cat#69077-3
pVV16	Vilchèze C et al.^65^	N/A
pMV306	Stover C.K et al.^66^	N/A
pMV261	Stover C.K et al.^66^	N/A

Software and Algorithms		

Prism 9.0	Graphpad	https://www.graphpad.com
Zen (Blue edition)	Zeiss	https://www.zeiss.com/microscopy/int/products/microscope-software/zen.htmL

Experimental Models: Cell Lines		

THP-1 macrophages	This paper	ATCC® TIB-202™
A549	This paper	ATCC® CCL-185™
A549-CD81-LEL-KO	This paper	N/A
Primary human monocyte-derived macrophages	Etablissement Français du sang (EFS), France	N/A

**Antibodies**		

Rat anti-HA (3F10)	Merck	Cat#11867423001; RRID:AB_390918
Rat anti-KasA	Viljoen A et al.^67^	N/A
Mouse anti-His	Merck	Cat#H1029-.2ML; RRID:AB_260015
Rabbit anti-BSA	Fisher Scientific	Cat#10092782
Rabbit anti-CD81	Abcam	Cat#ab109201; RRID:AB_10866464
Rabbit anti-Actin	Abcam	Cat#ab179467; RRID:AB_2737344
Mouse anti-GFP	Antibodies-online	Cat#ABIN387748
Mouse anti-HU CD43	Becton Dickinson	Cat#555474; RRID:AB_2638919
Mouse anti-HU CD82	Antibodies-online	Cat#ABIN1383874
Mouse anti-HU CD151	Becton Dickinson	Cat#556056; RRID:AB_2646510
Mouse anti-HU CD206/MR	Becton Dickinson	Cat#555953; RRID:AB_2649408
Mouse anti-HU CR3/CD11B	Life Technologies	Cat#4-0118-82
Mouse anti-HU CD81	Becton Dickinson	Cat#555675; RRID:AB_396028
Mouse anti-HU CD9	Becton Dickinson	Cat#555370; RRID:AB_395772
Mouse anti-HU CD63	Becton Dickinson	Cat#556019; RRID:AB_2646310
Mouse IgG1	Becton Dickinson	Cat#555746; RRID:AB_2648489
Alexa Fluor 488 anti-Mouse IgG (Goat)	Life Technologies	Cat#A11017; RRID:AB_2758366
Alexa Fluor 594 anti-Mouse IgG (Goat)	Life Technologies	Cat#A11032; RRID:AB_2534091
Anti-Rabbit IgG H&L (HRP) (Goat)	Abcam	Cat#ab205718; RRID:AB_2819160
Anti-mouse IgG H&L (HRP) (Goat)	Abcam	Cat#ab205719; RRID:AB_2755049
Anti-Rat IgG H&L (HRP) (Goat)	Abcam	Cat#ab97057; RRID:AB_10680316
Alexa Fluor 647 anti-Mouse IgG (Donkey)	ThermoFisher Scientific	Cat#A32787; RRID:AB_2762830

Chemicals, Peptides, and Recombinant Proteins		

Hydrogen peroxide	Merck	Cat#107209
Anhydride acetic acid	Merck	Cat#691275
Acetonitrile	Merck	Cat#34851
Trifluoroacetic acid	Merck	Cat#302031

Other		

Glutathione Sepharose 4FF, 25 ml	Sigma	Cat#GE17-5132-01
NI Sepharose 6FF, 25 ml	Sigma	Cat#GE17-5318-01
HA-Tag IP/Co-IP	Thermo Fisher Scientific	Cat#26180
GFP-Trap Agarose	Chromotek	Cat#gta-20
Latex beads, carboxylated, fluorescent yellow-green	Sigma	Cat#L4655-1ML
Phosphate Buffer Saline	Euromedex	Cat#ET330
Bovine Serum Albumin	Sigma	Cat#A0281

### Resource availability

#### Lead contact

Further information and requests for resources and reagents should be directed to and will be fulfilled by the lead contact, Wassim Daher (wassim.daher@irim.cnrs.fr).

#### Materials availability

All unique/stable reagents generated in this study are available from the lead contact with a completed Materials Transfer Agreement.

### Method details

#### Mycobacterial strains, growth conditions and reagents

All bacterial strains are listed in key resources table. Smooth (S) variant of *Mab* CIP104536^T^ was typically grown in Middlebrook 7H9 broth (BD Difco) supplemented with 0.05% Tween 80 and 10% oleic acid, albumin, dextrose, catalase (OADC enrichment; BD Difco) (7H9^T/OADC^) at 37°C in the presence of antibiotics, when required. Electrocompetent mycobacteria were transformed using a Bio-Rad Gene pulser (25 μF, 2500 V, 800 Ohms). For bacterial selection, media were supplemented either with 1 mg/ml hygromycin for strains carrying pTEC27 (Addgene, plasmid 30182), allowing tdTomato expression, and pMV306-*ahpCi* or with 250 μg/ml kanamycin when harboring the pMV306 or pMV261 or pVV16 derivatives. On plates, colonies were selected either on Middlebrook 7H10 agar (BD Difco) supplemented with 10% OADC enrichment (7H10^OADC^) or on LB agar.

#### Preparation of *Mab* inocula

Briefly, exponentially growing bacteria were collected by centrifugation and resuspended in 1 ml PBS. Bacterial suspensions were homogenized with a 26-G needle (15 up-and-down sequences) and sonicated three times for 10 s (with 10 s breaks between each sequence) in a water bath sonicator. 50 ml 7H9 were added to the suspended bacteria and filtered through 5 μm filters and bacteria were harvested after centrifugation at 3,000 x g for 5 min and finally resuspended in 200 μl 7H9-OADC. Before proceeding with the infection experiments, the final bacterial concentration was assessed by plating serial dilutions onto LB agar and CFU counting after 4 days of incubation at 37°C. Frozen inocula stocks (10 μl aliquots) were stored at −80°C. For each experiment, CFU were assessed from aliquots frozen at −80°C, a prerequisite to determine the exact number of viable bacteria to allow for reproducible use of bacteria from one experiment to another.

#### Construction of an *ahpC* repressible construct in *M. abscessus*

To control the expression level of *ahpC*, we adapted a repressible expression vector based on the tetOFF system.^41^^,^^68^ Briefly, we introduced the T38 sequences (encoded by *tetR38* with the *Ptb38* promoter sequence) and the *tet* operator sequence (*tetO-4C5G*) in the pMV306. This plasmid was used as a template for PCR amplification (primers 9/10) to insert the *ahpC* gene fused to a *ha*-tag. Subsequently, the *ahpC* gene (*MAB_4408c*) was amplified by PCR (primers 11/12), and then the two linear DNA fragments (vector and *ahpC*) were purified and circularized together using the In-Fusion HD cloning kit. The resulting plasmid pMV306-*ahpCi* was transformed into Stellar recipient cells, verified by sequencing, and finally introduced by electroporation into the *Mab* smooth morphotype strain.

#### Generation of the *ahpC* conditional knock-out mutant (*ΔahpCe/ahpCi-ha*)

The suicide vector pUX1-*katG* was used to generate unmarked single deletion mutant in *Mab* CIP104536^T^ (S). Briefly, the left and right arms (LA and RA, respectively) were PCR-amplified using genomic DNA and Q5 polymerase (New England Biolabs) as well as primers 1/2 (LA) and primers 3/4 (RA) (Table S1). The purified LA and RA amplicons were restricted with PacI/MfeI and EcoRI/NheI, respectively, and ligated to the PacI/NheI-linearized pUX1-*katG*, yielding pUX1-*katG-ahpC,* designed to delete 555 bp (94%) of the *ahpC* open reading frame. Electrocompetent *ahpCi Mab* strain was transformed with pUX1-*katG-ahpC* to generate the *ΔahpCe/ahpCi-ha* mutant strain. The selection of bacteria having undergone the first homologous recombination event was done by visual screening of red fluorescent colonies on 7H10^OADC^ supplemented with 250 μg/ml kanamycin and 1 mg/ml hygromycin. After subculturing the culture overnight in 7H9^T/OADC^ in the absence of kanamycin, bacterial suspensions were serially diluted and plated onto 7H10^OADC^ with 50 μg/ml INH and 1 mg/ml hygromycin to select for INH-resistant, Kan-sensitive and non-fluorescent colonies. The DNA junctions were PCR sequenced to confirm the genotype of the appropriate mutant using primers listed in Table S1. A representative *ΔahpCe*/*ahpCi-ha* clone was used for phenotypic analysis. Depletion of AhpCi-HA in the absence of ATc was assessed by Western blotting using anti-HA antibodies.

#### *In vitro* growth assays

Growth was inspected by inoculating the mid-log phase cultures into fresh 7H9 at an OD_600_ of 0.05. Cultures were incubated at 37°C ± ATc with shaking and OD_600_ was monitored for 96 h using a Synergy H1 hybrid reader (BioTek). For spot assays, 10-fold serial dilutions were made from exponentially grown cultures (37°C with agitation) in the absence of ATc. 1.5 μl of each dilution were spotted onto LB agar supplemented or not with 1 μg/ml ATc and plates incubated for 5 days at 37°C.

#### Invasion assay of *ΔahpCe/ahpCi-ha* in macrophages

Bacteria were pre-treated with 1 μg/ml ATc for 72 h and used to infect THP-1 cells for 3 h before being washed three times with 1x PBS and further incubated for 2 h with RPMI^FBS^ supplemented with 250 μg/ml amikacin to kill extracellular bacteria. Amikacin-containing media was then removed and cells were rinsed three times with 1x PBS. Macrophages were lysed with 100 μl of 1% Triton X100 and lysis was halted by adding 900 μl PBS. Successive dilutions of the suspension were plated on LB agar and incubated for 5 days at 37°C prior to CFU counting.

#### Overexpression of AhpC and Fmt in *M. abscessus*

PCR amplification of *ahpC* or *fmt* in fusion with an HA tag was performed using genomic DNA and the forward primers (13; HindIII and 15; MscI) and reverse primers (14; HpaI and 16; EcoRI). The amplicons were digested either with HindIII/HpaI or MscI/EcoRI and ligated into the HindIII/HpaI- or MscI/EcoRI-restricted pMV261 to generate pMV261-*ahpC*-HA or pMV261-*fmt*-HA, respectively. All constructs were sequenced and introduced in *Mab*.

#### Construction of *hsp60-Cd81-LEL-gfp*-expressing plasmid

The *Cd81-LEL* was PCR-amplified using the primers sets 17/18 (Table S1), *Homo sapiens* cDNA as template, and Q5 polymerase. The resulting amplicon was digested with NdeI/BamHI and subsequently ligated to NdeI-BamHI-linearized pVV16-GFP to produce an in-frame C-terminal fusion with GFP in the resulting plasmid, pVV16-*hsp60*-*Cd81-LEL*-*gfp*.

#### Western blotting

Bacteria were harvested, resuspended in PBS, and disrupted by bead beating using 1 mm diameter glass beads. Protein concentration was assessed using the BCA Protein Assay Reagent kit, according to the manufacturer’s instructions. Equal amounts of proteins (50 μg) were separated by SDS-PAGE and transferred to a nitrocellulose membrane. For detection of AhpC-HA, Fmt-HA, and KasA (loading control), membranes were probed for 1 h with rat anti-HA or rat anti-KasA antibodies^67^ (dilution 1:2,000). After washing, membranes were incubated for 45 min with goat anti-rat antibody conjugated to HRP (dilution 1:5,000) and bands were revealed using the ChemiDoc MP system (Bio-Rad laboratories). Similarly, for detection of GFP or CD81-LEL-GFP, and HSP16.7-His or AhpC-His or GroEL1, nitrocellulose membranes were probed for 1 h with mouse anti-GFP antibodies (dilution 1:2,000) and mouse anti-His antibodies (dilution 1:1,000). Anti-GFP and anti-His antibodies were diluted in 5% non-fat milk powder in TNT buffer (50 mM Tris pH 8; 150 mM NaCl; and 0.05% Tween 20). After washing, membranes were incubated for 45 min with goat anti-mouse antibodies conjugated to HRP (dilution 1:5,000). The GFP and His signals were revealed using a ChemiDoc MP system for imaging and analyzing gels. Likewise, for detection of BSA, nitrocellulose membranes were probed for 1 h with rabbit anti-BSA (dilution 1:5,000) antibody.

#### Identification of CD81-LEL-GFP protein partners

Briefly, 400 ml of a mycobacterial culture (for each strain) was grown to mid-exponential phase (OD_600_∼1.0) in 7H9 medium at 37°C with shaking. Cells were collected by centrifugation for 30 min at 10,000 × *g* and the pellet was washed once with PBS containing 1 mM benzamidine and then resuspended in 2 ml of PBS containing 1 mM benzamidine. Subsequently, the bacteria were lysed in a bead beater. The supernatants were collected by centrifugation for 10 min at 5,000 × *g*, and the resulting pellets were incubated in lysis buffer (50 mM Tris HCl pH 7.5, 250 mM NaCl, 5 mM EDTA, 1% NP40, 1% Triton, 1% Bridj96 and protease inhibitor [Benzamidine]) and then incubated on ice for 1 h. After centrifugation at 14,000 rpm during 45 min at 4°C, the pooled supernatants were subjected to immunoprecipitation using anti-GFP lama antibodies (GFP-Trap Agarose Beads). Following stringent washing conditions (25 mM Tris HCl pH 7.5, 150 mM NaCl, 0.025% Tween and protease inhibitor [Benzamidine]), one fifth of the beads for each condition were suspended in loading buffer for SDS-PAGE, and the proteins were then separated on a 4–12% gradient gel and detected by silver staining. The remaining beads were directly submitted to trypsin treatment for mass spectrometry analysis.

#### Mass spectrometry

After denaturation at 100°C in 5% SDS, 5% β-mercaptoethanol, 1-mM EDTA, 10% glycerol, and 10-mM Tris pH 8 buffer for 3 min, protein samples were fractionated on a 10% acrylamide SDS-PAGE gel. The electrophoretic migration was stopped as soon as the protein sample entered 6 mm into the separating gel. The gel was briefly labeled with Coomassie Blue, and three bands, containing the whole sample, were cut. In gel, digestion of gel slices was performed as previously described.^69^ An UltiMate 3000 RSLC nano System (Thermo Fisher Scientific) was used for separation of the protein digests. Peptides were processed and identified, as previously described.^70^

#### Proteomic data analysis

MS/MS data were analyzed using search engine Mascot (version 2.4.0, Matrix Science, London, UK). Searches were performed with a tolerance on mass measurement of 10 ppm for precursor and 0.02 Da for-fragment ions, against a composite target-decoy database (181,602∗2 total entries) built with a *Mycobacterium abscessus* Uniprot database (taxonomy 561007, march 2020, 4,940 entries). For each sample, peptides were filtered out according to the cut-off set for protein hits with one or more peptides taller than 9 residues, and a 1% false positive rate.

#### Subcellular fractionation of *M. abscessus*

Briefly, 400 ml of a mycobacterial culture were grown to middle exponential phase (OD_600_∼1.0) in 7H9 medium (containing glycerol, glucose and tyloxapol as described above) at 37°C with shaking. Cells were collected by a 30 min centrifugation at 10, 000 × *g*, and the pellet was washed once with PBS containing 1 mM benzamidine, and then re-suspended in 4 ml PBS containing 1 mM benzamidine. Subsequently, the bacteria were lysed in a bead beater and the resulting lysates transferred to 15 ml tubes and then subjected to two rounds of sonication for 30 s on ice. Lysates were centrifuged twice at 4, 000 × *g* for 10 min to remove unbroken cells. A 400 μl aliquot of each supernatant (serving as total lysate sample) was immediately removed and stored. The remaining supernatants were centrifuged at 16, 000 × *g* for 30 min at 4°C to pellet cell wall fraction. The supernatants were collected and ultra-centrifuged at 200, 000 × *g* for 2 h at 4°C to separate plasma membranes (pellet) from the cytoplasmic fraction. Cell wall and plasma membrane fractions were washed twice with PBS supplemented with 1 mM benzamidine and resuspended in 500 μl of PBS containing protease inhibitors. Cytoplasmic fractions were ultra-centrifuged one more time to discard all residual membranes.

#### Cloning of *hsp16.7* and *ahpC* genes in pET30

*hsp16.7* and *ahpC* genes were PCR-amplified from *Mab* CIP104536^T^ purified genomic DNA using the primers 23/24 and 25/26 (Table S1) and Q5 polymerase. The amplicons were cloned into pET30 restricted with EcoRI and KpnI, enabling the introduction of both genes in frame with the poly-histidine tag.

#### Expression and purification of His-tagged Hsp16.7 and AhpC recombinant proteins

pET30 constructs containing the *hsp16.7* or *ahpC* genes were used to transform *E. coli* strain BL21 Rosetta 2 (DE3) (Novagen). Cultures were grown in Luria-Bertani (LB) medium containing 200 μg/ml ampicillin and 30 μg/ml chloramphenicol until an optical density at 600 nm (OD_600_) of 0.8 was achieved. Liquid cultures were then placed on ice water for 30 min before addition of 1 mM isopropyl β-D-1-thiogalactopyranoside (IPTG) and incubation for another 20 h at 16°C. Bacteria were then collected by centrifugation (6, 000 × *g*, 4°C, 60 min) and pellets were resuspended in lysis buffer (50 mM Tris-HCl pH 8, 200 mM NaCl, 20 mM imidazole, 5 mM β-mercaptoethanol, 1 mM benzamidine). Cells were lysed by sonication and the lysate clarified by centrifugation (28, 000 × *g*, 4°C, 45 min) and subjected to nickel affinity purification using Ni-NTA Sepharose beads according to the manufacturer’s instructions. After several washes, both proteins were eluted and then dialyzed overnight at 4°C in buffer containing 50 mM Tris-HCl pH 8, 200 mM NaCl and 5 mM β-mercaptoethanol. Purified proteins were assessed by SDS-PAGE and Coomassie Blue staining.

#### Expression and purification of recombinant CD81-LEL-GST and CD151-LEL-GST

CD81-LEL and CD151-LEL encoding sequences were amplified from *H. sapiens* cDNA using primers 19/20 and 21/22 and cloned into the pGEX-2T (gift from Dr Eric Rubinstein) using the BamHI/BglII and EcoRI sites to incorporate a GST tag at the C-terminus. The resulting plasmids were introduced into *E. coli* BL21 Rosetta 2 (DE3) (Novagen) for protein expression. Recombinant proteins were purified on Glutathione Sepharose 4 Fast Flow according to the manufacturer’s instructions. Purified proteins were assessed by SDS-PAGE and Coomassie Blue staining.

#### Macrophage and A549 infection assays

THP-1 macrophages were grown in RPMI medium supplemented with 10% fetal bovine serum (FBS; Sigma-Aldrich) and incubated at 37°C with 5% CO_2_. THP-1 monocytes were differentiated in macrophages in the presence of 20 ng/ml phorbol myristate acetate in 24-well flat-bottom tissue culture microplates (10^5^ cells/well) and incubated for 48 h at 37°C with 5% CO_2_. A549-WT and A549-CD81-LEL-KO were grown in DMEM supplemented with 10% FBS and incubated at 37°C with 5% CO_2_. Infection with *Mab* carrying pTEC27 (MOI 2:1) was performed for 3 h at 37°C in 5% CO_2_. Cells were carefully washed three times with 1× PBS and then incubated for 2 h with RPMI^FBS^ supplemented with 250 μg/ml amikacin to kill extracellular bacteria. The medium containing amikacin was then discarded and cells were washed three times with 1× PBS. To assess CFU, macrophages were lysed with 100 μl of 1% Triton X100. Lysis was stopped by adding 900 μl PBS and serial dilutions were plated to monitor the intracellular bacterial counts. CFU were counted after 5 days of incubation at 37°C.

#### Infection of primary human monocyte-derived macrophages and intracellular CFU determination

Buffy coats from anonymous donors were obtained from the Etablissement Français du Sang (EFS), Montpellier, France. Peripheral blood mononuclear cells (PBMCs) were purified by Ficoll density gradient separation. Monocytes were isolated from PBMCs using a magnetic cell separation system with anti-CD14 mAb-coated microbeads and then cultured in complete IMDM supplemented with 50 ng/mL GM-CSF (every 2 days) at 37°C under a humidified 5% CO2 atmosphere for 6 days. Cells were washed and detached with 20 mM EDTA in PBS and plated in a 24-well plate at a concentration of 10^5^ cells/well with complete IMDM. Monocyte-derived macrophages were infected with *Mab* for 3 h at a MOI of 2:1, washed, treated with 250 μg/mL amikacin for 2 h and replenished with fresh medium. To determine the number of internalized bacilli at 3 hpi, cells were lysed with 100 μl of 1% Triton X100 and lysates plated on LB agar plates prior to CFU counting.

#### Immunofluorescence staining of *Mab*-infected cells

For microscopy-based infectivity assays, THP-1 and A549 cells were cultured on coverslips in 24-well plates at a density of 10^5^ cells/well. Cells were infected with tdTomato-expressing *Mab* (MOI 2:1) for 3 h, washed, treated with amikacin, fixed at 3 hpi with 4% paraformaldehyde in PBS for 20 min and then permeabilized using 0.2% Triton X-100 for 20 min. After blocking with 2% BSA in PBS supplemented with 0.2% Triton X-100 for 20 min, cells were incubated with anti-CD43 antibody (dilution 1:1,000) for 1 h and with an Alexa Fluor 488- or 594-conjugated anti-mouse secondary antibody. Cells were then stained with 1 μg/ml 4′,6-diamidino-2-phenylindole (DAPI) for 5 min, washed with PBS, mounted onto microscope slides using Immumount (Calbiochem) and examined with either an epifluorescence microscope using a 63x lens objective or with a confocal microscope (Zeiss LSM880) using a 40× objective. Quantification of the percentage of host cells containing bacilli was done using an epifluorescence microscope. The average proportion of macrophages containing fewer than < 5, 5−10, or > 10 bacteria was quantified using Zeiss Axiovision software. Images were acquired by focusing on combined signals (CD43 in green and red fluorescent *Mab*) and captured on a Zeiss Axioimager confocal microscope equipped with a 40x or 63x oil objective and processed using the Zeiss Axiovision software. Scoring the number of bacilli present within macrophages was performed using ImageJ. Equal parameters for the capture and scoring of images were consistently applied to all samples.

#### HA-trap pull-down experiments

*Mab* expressing GFP, GFP-tagged CD81-LEL, or HA-tagged AhpC were harvested in 400 ml of liquid medium (7H9^OADC^ and 0.025% tyloxapol). Pellets from mid-log phase cultures were collected and resuspended in 2 ml of PBS with benzamidine (1 mM protease inhibitor). Bacterial pellets were broken using a bead beater twice for 3 min (30 beats/s) and incubated on ice after each cycle. After centrifugation, supernatants containing bacterial cytosolic proteins were collected. The remaining pellets were then suspended with 500 μl of lysis buffer containing 3 detergents (50 mM Tris-HCl pH= 7.5, 250 mM NaCl, 5 mM EDTA, 1% NP-40, 1% Triton, 1% Bridge96, 1 mM Benzamidine). Samples were then incubated on a rotary mixer at 4°C for 2 h and centrifugated at 13, 500 rpm for 5 min at 4°C. The supernatants were collected and contained the membrane-associated proteins. Subsequently, the two supernatants were mixed and then incubated with 40 μl of anti-HA agarose beads. The samples were incubated on rotary mixer at 4°C overnight. The next day, the samples were centrifuged and the supernatant was discarded. Anti-HA beads were collected in the pellet and washed twice with TBS-T pH 7.2 (25 mM Tris-HCl, 0.15 M NaCl, 0.025% Tween). The beads were boiled with Laemmli sample buffer to extract bounded proteins and then loaded on 12% SDS-page for Western blotting.

#### Coating of latex beads with recombinant proteins

Fifty μl of 1 μm diameter carboxylated fluorescein-labeled latex beads (Sigma-Aldrich) were washed with PBS and incubated with 1 mg/ml purified proteins (AhpC-His, Hsp16.7-His or BSA) at room temperature on a rotary mixer overnight. The beads were then washed to remove unbound proteins, followed by saturation with 10 mg/ml BSA for 1 h at room temperature. Finally, the latex beads were suspended with 1ml RPMI containing 10% FBS to be used for internalization assays. The beads were boiled with Laemmli sample buffer to extract bound proteins, which were separated on a 12% SDS-PAGE. Additional analyses of the proteins attached to the latex beads were performed by immunofluorescence microscopy. Beads were first incubated with mouse anti-His or rabbit anti-BSA primary antibodies, washed and incubated with Alexa Fluor 594-conjugated goat anti-mouse or goat anti-rabbit antibodies, respectively. Fluorescence images were generated using a confocal microscope.

#### Bead phagocytosis assay

Protein-coated beads were added to the macrophages at a concentration of 10 beads/cell. After 4 h of incubation at 37°C, macrophages were washed five times with PBS before labeling using anti-CD43 antibodies and Alexa Fluor 594-coupled anti-mouse secondary antibodies. Quantification of the percentage of macrophages containing beads was done using an epifluorescence microscope.

#### Antibody inhibition assays in macrophages

Antibody inhibition experiments were performed after pre-incubating the THP-1 or HMDM cells with the specific antibodies in RPMI or DMEM containing FBS for 3 h at a concentration of 50 μg/mL before determining the intracellular bacterial loads (CFUs) and the percentage of bead-containing macrophages.

#### Peptides synthesis

All peptides (100 micromoles) were synthesized by solid phase at the SynBio3 platform (Institute of Biomolecule Max Mousseron (IBMM), Montpellier, France) using the conventional Fmoc chemistry performed on a Liberty Blue microwave peptide synthesizer (CEM, Matthews, NC, USA). The used resin (AmphiSphereTM, Agilent, Les Ullis, France) provides an amide C-terminal end for the peptides. Before deprotection and cleavage of the peptides from the resin, the N-terminal amine function of each peptide was acetylated by a treatment with anhydride acetic acid as commonly performed in solid phase peptide synthesis (SPPS). These N- and C-terminal modifications induce the neutralization of the cationic and anionic charges normally present at these peptide extremities. Therefore, potential interactions of these peptides with any protein are not impaired by ionic repulsions. Following cleavage and deprotection of the N-acetylated peptides, crude peptides were purified by reversed-phase high-performance liquid chromatography (HPLC) on a semi-preparative system with an increased acetonitrile gradient. Homogeneous fractions for each peptide were pooled and freeze-dried. A qualitative analysis of each peptide was assessed by analytical HPLC and electrospray ionization mass spectrometry. All peptides were purified with a good purity (>90%) and mass spectrometry values corresponded to the expected mass values.

For peptide oxidation, the dried peptides were resuspended in a volume of water containing 0.1% trifluoroacetic acid (TFA) at the concentration of 1 mM. This level of TFA allows to maintain the peptide in an acidic environment and to avoid the oxidation of the peptide. In general, half of the peptide volume was then aliquoted, frozen and freeze-dried until further use. The other half was submitted to the oxidation of the cysteine as follows: first, the pH was brought to a slight alkaline pH (pH 8) upon the addition of 10% volume/volume of Tris-HCl 500 mM, pH 8. Oxidation was then initiated by the addition of 5% vol/vol of hydrogen peroxide in the peptide solution. At this concentration (1 mM and below), the intramolecular oxidation of the peptide is favored. At a higher concentration, cysteine oxidation could occur intermolecularly, leading thus to the polymerization of the peptide through the formation of disulfide bridge between several molecules of peptide. The cyclisation step could be easily monitored by analytical HPLC and generally occurred within a couple of hours. Finally, peptide solutions were submitted to LC-MS analysis and the molecular masses were in agreements with the oxidation process with a lower mass detected for the oxidized form compared to the reduced one. Both reduced and oxidized peptides were then aliquoted, frozen and freeze-dried until further use.

#### Characteristics of the peptides used in this study

##### CD81-810S peptide (19 AA): C_157_GSSTLTALTTSVLKNNLC_175_

Reduced form: CD81-810SH Peptide calculated mass: 1967.30 g/mol; found: 1967.39 g/mol.

Oxidized form: CD81-810SS Peptide calculated mass: 1965.30 g/mol; found: 1966.41 g/mol.

##### CD81-910S peptide (16 AA): C_175_PSGSNIISNLFKEDC_190_

Reduced form: CD81-910SH Peptide calculated mass: 1768.00 g/mol; found: 1768.80 g/mol.

Oxidized form: CD81-910SS Peptide calculated mass: 1766.00 g/mol; found: 1765.47 g/mol.

##### Scrambled sequence: C_175_SNLGIESDFPINKSC_190_

Reduced form: CD81-910 SCR SH Peptide calculated mass: 1768.00 g/mol; found: 1767.64 g/mol.

Oxidized form: CD81-910 SCR SS Peptide calculated mass: 1766.00 g/mol; found: 1764.96 g/mol.

#### Saturating *M. abscessus* AhpC with recombinant CD81-LEL or derived peptides

*Mab* was first saturated for 3 h with either the soluble GST-LEL fusion proteins or with CD81-LEL-derived peptides at different concentrations before infecting human macrophages for an additional 3 h. Alternatively, to saturate the endogenous CD81 expressed by the host cells, macrophages were pre-incubated with the recombinant AhpC-His before adding the whole bacilli. Microscopic images were then acquired and CFUs were quantified as described above.

#### Flow cytometry

Cells were harvested in PBS/50 mM EDTA, washed in PBS and fixed with 2% paraformaldehyde. For cell surface expression analysis, cells were stained in PBS/1% BSA with indicated primary antibodies at 4°C for 30 min followed by three washes and incubation for 25 min with diluted secondary antibodies. For intracellular staining, cells were permeabilized in a PBS/1% BSA/0.025% saponin solution for 15 min prior to staining with corresponding primary antibodies diluted in the permeabilization solution for 45 min at 4°C. Analyses were done using either FlowJo software (Treestar Inc., Oregon, USA) or NovoExpress software (Agilent, California, USA).

#### CRISPR/Cas9-mediated disruption of *Cd81-LEL* in A549 cells

For CRISPR/Cas9-mediated disruption of *Cd81-LEL*, A549 cells stably expressing Cas9 were generated by transduction with the LentiCas9-Blast vector followed by selection with blasticidin at 10 μg/ml. ^35^ A549 cells expressing Cas9 were transduced with the viral particles generated in HEK-293T cells. Viral particles were produced by standard polyethylenimine transfection of HEK293T monolayers with three different vectors (p8.91, VSV-G, and the tetraspanin-*Cd81-LEL* LentiGuide-Puro vector containing tetraspanin-*Cd81-LEL-*specific guide RNA) at a ratio of 1/1/0.5. *Cd81-LEL* guide RNA-encoding oligonucleotides (27 and 28, Table S1) were annealed and ligated into the BsmBI-digested LentiGuide-Puro vector. The guide was designed to target the fourth coding exon of the *Cd81* open reading frame upstream of the *LEL* nucleotide sequence. Viral particles were collected 36 h post-transfection, filtered and used directly to transduce A549-Cas9 cells at high multiplicity of infection. After two days, cells were selected with puromycin for 10-15 days. Single CRISPR/Cas9 *Cd81-LEL*-knock-out clones were isolated by flow cytometry and disruption of *Cd81-LEL* was verified by PCR/sequencing of the *Cd81* open reading frame.

### Quantification and statistical analysis

All data are represented as mean ± S.D. as indicated in the corresponding figure legends. The ‘n’ value represents either the number of biological replicates or the number of independent experiments (or as specified otherwise) and are indicated in each figure legend. Statistical analysis was performed using the Prism 9.0 software (Graphpad, USA). For the comparison of two independent datasets, two-tailed unpaired Student’s t tests were applied, assuming equal variance. Multiple datasets were statistically compared *via* one- or two-way analysis of variance (ANOVA). The p value was considered significant if < 0.05 and significance was indicated as follows: ∗p < 0.05, ∗∗p < 0.01, ∗∗∗p < 0.001, ∗∗∗∗p < 0.0001.

## Data Availability

• Data reported in this paper will be shared by the lead contact upon request. • This study did not generate any unique datasets or code. • Any additional information required to reanalyze the data reported in this paper is available from the lead contact upon request.

## References

[1] Pereira A.C., Ramos B., Reis A.C., Cunha M.V. (2020). Non-tuberculous mycobacteria: molecular and physiological bases of virulence and adaptation to ecological niches. Microorganisms.

[2] Sharma S.K., Upadhyay V. (2020). Epidemiology, diagnosis & treatment of non-tuberculous mycobacterial diseases. Indian J. Med. Res..

[3] Ratnatunga C.N., Lutzky V.P., Kupz A., Doolan D.L., Reid D.W., Field M., Bell S.C., Thomson R.M., Miles J.J. (2020). The rise of non-tuberculosis mycobacterial lung disease. Front. Immunol..

[4] Hannah C.E., Ford B.A., Chung J., Ince D., Wanat K.A. (2020). Characteristics of non-tuberculous mycobacterial infections at a midwestern tertiary hospital: a retrospective study of 365 patients. Open Forum Infect. Dis..

[5] Johansen M.D., Herrmann J.-L., Kremer L. (2020). Non-tuberculous mycobacteria and the rise of *Mycobacterium abscessus*. Nat. Rev. Microbiol..

[6] To K., Cao R., Yegiazaryan A., Owens J., Venketaraman V. (2020). General overview of non-tuberculous mycobacteria opportunistic pathogens: *Mycobacterium avium* and *Mycobacterium abscessus*. J. Clin. Med..

[7] Dubois V., Pawlik A., Bories A., Le Moigne V., Sismeiro O., Legendre R., Varet H., Rodríguez-Ordóñez M.D.P., Gaillard J.-L., Coppée J.Y. (2019). *Mycobacterium abscessus* virulence traits unraveled by transcriptomic profiling in amoeba and macrophages. PLoS Pathog..

[8] Daher W., Leclercq L.-D., Johansen M.D., Hamela C., Karam J., Trivelli X., Nigou J., Guérardel Y., Kremer L. (2022). Glycopeptidolipid glycosylation controls surface properties and pathogenicity in *Mycobacterium abscessus*. Cell Chem. Biol..

[9] Kim B.-R., Kim B.-J., Kook Y.-H., Kim B.-J. (2019). Phagosome escape of rough *Mycobacterium abscessus* strains in murine macrophage via phagosomal rupture can lead to type I interferon production and their cell-to-cell spread. Front. Immunol..

[10] Kim B.-R., Kim B.-J., Kook Y.-H., Kim B.-J. (2020). *Mycobacterium abscessus* infection leads to enhanced production of type 1 interferon and NLRP3 inflammasome activation in murine macrophages via mitochondrial oxidative stress. PLoS Pathog..

[11] Le Moigne V., Roux A.-L., Jobart-Malfait A., Blanc L., Chaoui K., Burlet-Schiltz O., Gaillard J.-L., Canaan S., Nigou J., Herrmann J.-L. (2020). A TLR2-activating fraction from *Mycobacterium abscessus* rough variant demonstrates vaccine and diagnostic potential. Front. Cell. Infect. Microbiol..

[12] Malcolm K.C., Caceres S.M., Pohl K., Poch K.R., Bernut A., Kremer L., Bratton D.L., Herrmann J.-L., Nick J.A. (2018). Neutrophil killing of *Mycobacterium abscessus* by intra- and extracellular mechanisms. PLoS One.

[13] Ryan K., Byrd T.F. (2018). *Mycobacterium abscessus*: shapeshifter of the mycobacterial world. Front. Microbiol..

[14] Zhang C., Asif H., Holt G.E., Griswold A.J., Campos M., Bejarano P., Fregien N.L., Mirsaeidi M. (2019). *Mycobacterium abscessus*-bronchial epithelial cells cross-talk through type I interferon signaling. Front. Immunol..

[15] Roux A.-L., Viljoen A., Bah A., Simeone R., Bernut A., Laencina L., Deramaudt T., Rottman M., Gaillard J.-L., Majlessi L. (2016). The distinct fate of smooth and rough *Mycobacterium abscessus* variants inside macrophages. Open Biol..

[16] Shamaei M., Mirsaeidi M. (2021). Non-tuberculous mycobacteria, macrophages, and host innate immune response. Infect. Immun..

[17] Tran T., Bonham A.J., Chan E.D., Honda J.R. (2019). A paucity of knowledge regarding non-tuberculous mycobacterial lipids compared to the tubercle bacillus. Tuberculosis.

[18] Liu C.H., Liu H., Ge B. (2017). Innate immunity in tuberculosis: host defense vs pathogen evasion. Cell. Mol. Immunol..

[19] Upadhyay S., Mittal E., Philips J.A. (2018). Tuberculosis and the art of macrophage manipulation. Pathog. Dis..

[20] Lee H.-J., Woo Y., Hahn T.-W., Jung Y.M., Jung Y.-J. (2020). Formation and maturation of the phagosome: a key mechanism in innate immunity against intracellular bacterial infection. Microorganisms.

[21] Ribeiro G.M., Matsumoto C.K., Real F., Teixeira D., Duarte R.S., Mortara R.A., Leão S.C., de Souza Carvalho-Wodarz C. (2017). Increased survival and proliferation of the epidemic strain *Mycobacterium abscessus subsp. massiliense* CRM0019 in alveolar epithelial cells. BMC Microbiol..

[22] Karam J., Méresse S., Kremer L., Daher W. (2020). The roles of tetraspanins in bacterial infections. Cell Microbiol..

[23] Hemler M.E. (2008). Targeting of tetraspanin proteins--potential benefits and strategies. Nat. Rev. Drug Discov..

[24] Homsi Y., Schloetel J.-G., Scheffer K.D., Schmidt T.H., Destainville N., Florin L., Lang T. (2014). The extracellular δ-domain is essential for the formation of CD81 tetraspanin webs. Biophys. J..

[25] Huang Y., Yu L. (2022). Tetraspanin-enriched microdomains: the building blocks of migrasomes. Cell Insight.

[26] Kumar A., Hossain R.A., Yost S.A., Bu W., Wang Y., Dearborn A.D., Grakoui A., Cohen J.I., Marcotrigiano J. (2021). Structural insights into hepatitis C virus receptor binding and entry. Nature.

[27] Gordón-Alonso M., Yañez-Mó M., Barreiro O., Alvarez S., Muñoz-Fernández M.A., Valenzuela-Fernández A., Sánchez-Madrid F. (2006). Tetraspanins CD9 and CD81 modulate HIV-1-induced membrane fusion. J. Immunol..

[28] Silvie O., Rubinstein E., Franetich J.-F., Prenant M., Belnoue E., Rénia L., Hannoun L., Eling W., Levy S., Boucheix C., Mazier D. (2003). Hepatocyte CD81 is required for *Plasmodium falciparum* and *Plasmodium yoelii* sporozoite infectivity. Nat. Med..

[29] Tham T.N., Gouin E., Rubinstein E., Boucheix C., Cossart P., Pizarro-Cerda J. (2010). Tetraspanin CD81 is required for *Listeria monocytogenes* invasion. Infect. Immun..

[30] Astarie-Dequeker C., N’Diaye E.N., Le Cabec V., Rittig M.G., Prandi J., Maridonneau-Parini I. (1999). The mannose receptor mediates uptake of pathogenic and non-pathogenic mycobacteria and bypasses bactericidal responses in human macrophages. Infect. Immun..

[31] Fratazzi C., Manjunath N., Arbeit R.D., Carini C., Gerken T.A., Ardman B., Remold-O’Donnell E., Remold H.G. (2000). A macrophage invasion mechanism for mycobacteria implicating the extracellular domain of CD43. J. Exp. Med..

[32] Hetland G., Wiker H.G. (1994). Antigen 85C on *Mycobacterium bovis*, BCG and *M. tuberculosis* promotes monocyte-CR3-mediated uptake of microbeads coated with mycobacterial products. Immunology.

[33] Hickey T.B.M., Ziltener H.J., Speert D.P., Stokes R.W. (2010). *Mycobacterium tuberculosis* employs Cpn60.2 as an adhesin that binds CD43 on the macrophage surface. Cell Microbiol..

[34] Sendide K., Reiner N.E., Lee J.S.I., Bourgoin S., Talal A., Hmama Z. (2005). Cross-talk between CD14 and complement receptor 3 promotes phagocytosis of mycobacteria: regulation by phosphatidylinositol 3-kinase and cytohesin-1. J. Immunol..

[35] Doyle T., Moncorgé O., Bonaventure B., Pollpeter D., Lussignol M., Tauziet M., Apolonia L., Catanese M.-T., Goujon C., Malim M.H. (2018). The interferon-inducible isoform of NCOA7 inhibits endosome-mediated viral entry. Nat. Microbiol..

[36] Master S.S., Springer B., Sander P., Boettger E.C., Deretic V., Timmins G.S. (2002). Oxidative stress response genes in *Mycobacterium tuberculosis*: role of *ahpC* in resistance to peroxynitrite and stage-specific survival in macrophages. Microbiology (Read.).

[37] Kumar S., Puniya B.L., Parween S., Nahar P., Ramachandran S. (2013). Identification of novel adhesins of *M. tuberculosis* H37Rv using integrated approach of multiple computational algorithms and experimental analysis. PLoS One.

[38] Daher W., Leclercq L.-D., Viljoen A., Karam J., Dufrêne Y.F., Guérardel Y., Kremer L. (2020). O-methylation of the glycopeptidolipid acyl chain defines surface hydrophobicity of *Mycobacterium abscessus* and macrophage invasion. ACS Infect. Dis..

[39] Richard M., Gutiérrez A.V., Viljoen A., Rodriguez-Rincon D., Roquet-Baneres F., Blaise M., Everall I., Parkhill J., Floto R.A., Kremer L. (2019). Mutations in the MAB_2299c TetR regulator confer cross-resistance to clofazimine and bedaquiline in *Mycobacterium abscessus*. Antimicrob. Agents Chemother..

[40] Ehrt S., Guo X.V., Hickey C.M., Ryou M., Monteleone M., Riley L.W., Schnappinger D. (2005). Controlling gene expression in mycobacteria with anhydrotetracycline and Tet repressor. Nucleic Acids Res..

[41] Kim J.-H., O’Brien K.M., Sharma R., Boshoff H.I.M., Rehren G., Chakraborty S., Wallach J.B., Monteleone M., Wilson D.J., Aldrich C.C. (2013). A genetic strategy to identify targets for the development of drugs that prevent bacterial persistence. Proc. Natl. Acad. Sci. USA.

[42] Vinod V., Vijayrajratnam S., Vasudevan A.K., Biswas R. (2020). The cell surface adhesins of *Mycobacterium tuberculosis*. Microbiol. Res..

[43] Hantak M.P., Qing E., Earnest J.T., Gallagher T. (2019). Tetraspanins: architects of viral entry and exit platforms. J. Virol..

[44] Manzoni G., Marinach C., Topçu S., Briquet S., Grand M., Tolle M., Gransagne M., Lescar J., Andolina C., Franetich J.-F. (2017). Plasmodium P36 determines host cell receptor usage during sporozoite invasion. Elife.

[45] New C., Lee Z.-Y., Tan K.S., Wong A.H.-P., Wang D.Y., Tran T. (2021). Tetraspanins: host factors in viral infections. Int. J. Mol. Sci..

[46] van Spriel A.B., Figdor C.G. (2010). The role of tetraspanins in the pathogenesis of infectious diseases. Microb. Infect..

[47] Green L.R., Monk P.N., Partridge L.J., Morris P., Gorringe A.R., Read R.C. (2011). Cooperative role for tetraspanins in adhesin-mediated attachment of bacterial species to human epithelial cells. Infect. Immun..

[48] Raynaud C., Daher W., Johansen M.D., Roquet-Banères F., Blaise M., Onajole O.K., Kozikowski A.P., Herrmann J.-L., Dziadek J., Gobis K., Kremer L. (2020). Active benzimidazole derivatives targeting the MmpL3 transporter in *Mycobacterium abscessus*. ACS Infect. Dis..

[49] Asadi A., Razavi S., Talebi M., Gholami M. (2019). A review on anti-adhesion therapies of bacterial diseases. Infection.

[50] Robert J.-M.H., Amoussou N.G., Mai H.L., Logé C., Brouard S. (2021). Tetraspanins: useful multifunction proteins for the possible design and development of small-molecule therapeutic tools. Drug Discov. Today.

[51] Ventress J.K., Partridge L.J., Read R.C., Cozens D., MacNeil S., Monk P.N. (2016). Peptides from tetraspanin CD9 are potent inhibitors of *Staphylococcus aureus* adherence to keratinocytes. PLoS One.

[52] Hassuna N.A., Monk P.N., Ali F., Read R.C., Partridge L.J. (2017). A role for the tetraspanin proteins in Salmonella infection of human macrophages. J. Infect..

[53] Guimarães B.G., Souchon H., Honoré N., Saint-Joanis B., Brosch R., Shepard W., Cole S.T., Alzari P.M. (2005). Structure and mechanism of the alkylhydroperoxidase AhpC, a key element of the *Mycobacterium tuberculosis* defense system against oxidative stress. J. Biol. Chem..

[54] Israels S.J., McMillan-Ward E.M. (2007). Platelet tetraspanin complexes and their association with lipid rafts. Thromb. Haemostasis.

[55] Berditchevski F. (2001). Complexes of tetraspanins with integrins: more than meets the eye. J. Cell Sci..

[56] Kummer D., Steinbacher T., Schwietzer M.F., Thölmann S., Ebnet K. (2020). Tetraspanins: integrating cell surface receptors to functional microdomains in homeostasis and disease. Med. Microbiol. Immunol..

[57] Termini C.M., Gillette J.M. (2017). Tetraspanins function as regulators of cellular signaling. Front. Cell Dev. Biol..

[58] Yunta M., Lazo P.A. (2003). Tetraspanin proteins as organisers of membrane microdomains and signaling complexes. Cell. Signal..

[59] Suárez H., Rocha-Perugini V., Álvarez S., Yáñez-Mó M. (2018). Tetraspanins, another piece in the HIV-1 replication puzzle. Front. Immunol..

[60] Ye J. (2007). Reliance of host cholesterol metabolic pathways for the life cycle of hepatitis C virus. PLoS Pathog..

[61] Silvie O., Charrin S., Billard M., Franetich J.-F., Clark K.L., van Gemert G.-J., Sauerwein R.W., Dautry F., Boucheix C., Mazier D., Rubinstein E. (2006). Cholesterol contributes to the organization of tetraspanin-enriched microdomains and to CD81-dependent infection by malaria sporozoites. J. Cell Sci..

[62] Tsai Y.-H., Chen W.-L. (2020). Host lipid rafts as the gates for *Listeria monocytogenes* infection: a mini-review. Front. Immunol..

[63] Pawlik A., Garnier G., Orgeur M., Tong P., Lohan A., Le Chevalier F., Sapriel G., Roux A.-L., Conlon K., Honoré N. (2013). Identification and characterization of the genetic changes responsible for the characteristic smooth-to-rough morphotype alterations of clinically persistent *Mycobacterium abscessus*. Mol. Microbiol..

[64] Takaki K., Davis J.M., Winglee K., Ramakrishnan L. (2013). Evaluation of the pathogenesis and treatment of *Mycobacterium marinum* infection in zebrafish. Nat. Protoc..

[65] Vilchèze C., Molle V., Carrère-Kremer S., Leiba J., Mourey L., Shenai S., Baronian G., Tufariello J., Hartman T., Veyron-Churlet R. (2014). Phosphorylation of KasB regulates virulence and acid-fastness in *Mycobacterium tuberculosis*. PLoS Pathog..

[66] Stover C.K., de la Cruz V.F., Fuerst T.R., Burlein J.E., Benson L.A., Bennett L.T., Bansal G.P., Young J.F., Lee M.H., Hatfull G.F. (1991). New use of BCG for recombinant vaccines. Nature.

[67] Viljoen A., Blaise M., de Chastellier C., Kremer L. (2016). MAB_3551c encodes the primary triacylglycerol synthase involved in lipid accumulation in *Mycobacterium abscessus*. Mol. Microbiol..

[68] Schnappinger D., Ehrt S. (2014). Regulated expression systems for mycobacteria and their applications. Microbiol. Spectr..

[69] Miguet L., Béchade G., Fornecker L., Zink E., Felden C., Gervais C., Herbrecht R., Mauvieux L., van Dorsselaer A., Sanglier-Cianferani S. (2009). Proteomic analysis of malignant B-cell derived microparticles reveals CD148 as a potentially useful antigenic biomarker for mantle cell lymphoma diagnosis. J. Proteome Res..

[70] Berry L., Chen C.-T., Francia M.E., Guerin A., Graindorge A., Saliou J.-M., Grandmougin M., Wein S., Bechara C., Morlon-Guyot J. (2018). *Toxoplasma gondii* chromosomal passenger complex is essential for the organization of a functional mitotic spindle: a prerequisite for productive endodyogeny. Cell. Mol. Life Sci..

