# Supplementary material for: Mycobacterium abscessus alkyl hydroperoxide reductase C promotes cell invasion by binding to tetraspanin CD81
Source: iScience. 2023 Jan 25;26(2):106042. doi: 10.1016/j.isci.2023.106042 (PMC9929602; doi:10.1016/j.isci.2023.106042)
Supplement: Document S1. Figures S1–S7 and Table S1 [file mmc1.pdf]

iScience, Volume *26*

Supplemental information

Mycobacterium abscessus alkyl hydroperoxide reductase C promotes cell invasion by binding to tetraspanin CD81

Jona Karam, Fabien P. Blanchet, Éric Vivès, Prisca Boisguérin, Yves-Marie Boudehen, Laurent Kremer, and Wassim Daher

SUPPLEMENTAL INFORMATION

Supplemental Information includes 7 figures and 1 Table.

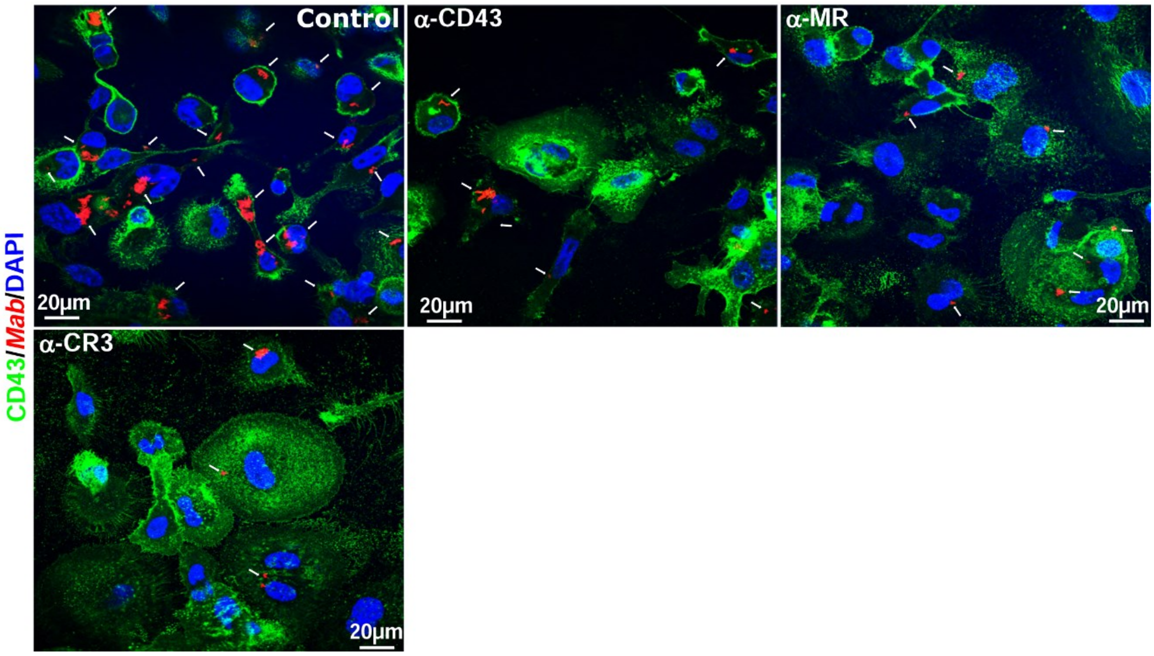

Supplementary Figure 1 - Efficient internalization of *Mab* by macrophages requires host CD43, MR and CR3 receptors, Related to Figure 1

Four immunofluorescent fields were taken at 3 h post-infection using a confocal microscope (40 x magnification), showing *Mab*-infected macrophages without or following pre-treatment with the indicated antibodies. The nuclei are shown in blue, the CD43 protein associated with the plasma membrane of macrophages is in green. White arrows indicate *Mab* (red) inside the macrophage.

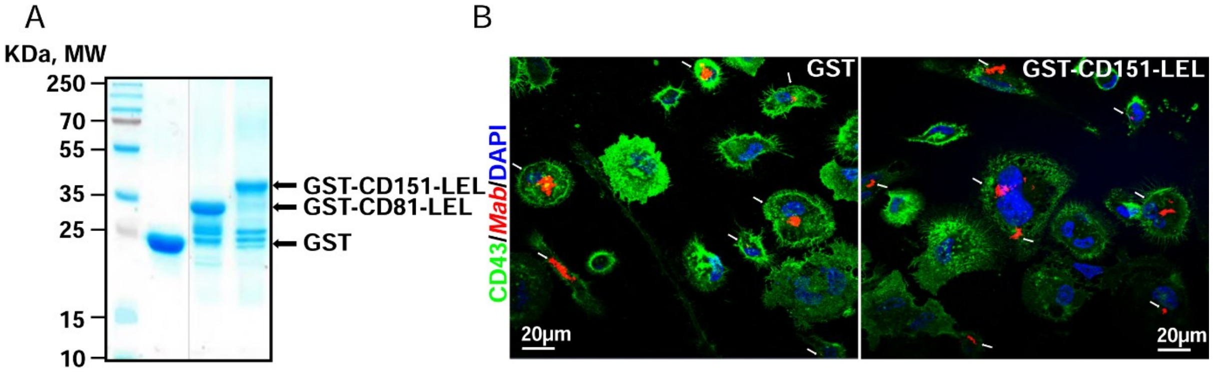

Supplementary Figure 2 - Recombinant GST-CD151-LEL fusion protein does not affect *Mab* uptake by macrophages, Related to Figure 2

(A) SDS-PAGE analysis of recombinant GST, GST-CD81-LEL or GST-CD151-LEL fusion proteins. Soluble proteins were purified from *E. coli*, separated on a 12% polyacrylamide gel and visualized with Coomassie brilliant blue. MW: protein molecular weight marker; GST: glutathione-*S*-transferase.

(B) Two immunofluorescent fields were captured using a confocal microscope (40 x magnification) 3 h after infection, showing *Mab*-infected macrophages after pre-treatment of bacteria with GST or GST-CD151-LEL. The nuclei are shown in blue, and the CD43 protein, which is associated with macrophage plasma membranes, is in green. *Mab* (in red) inside macrophages are indicated by white arrows.

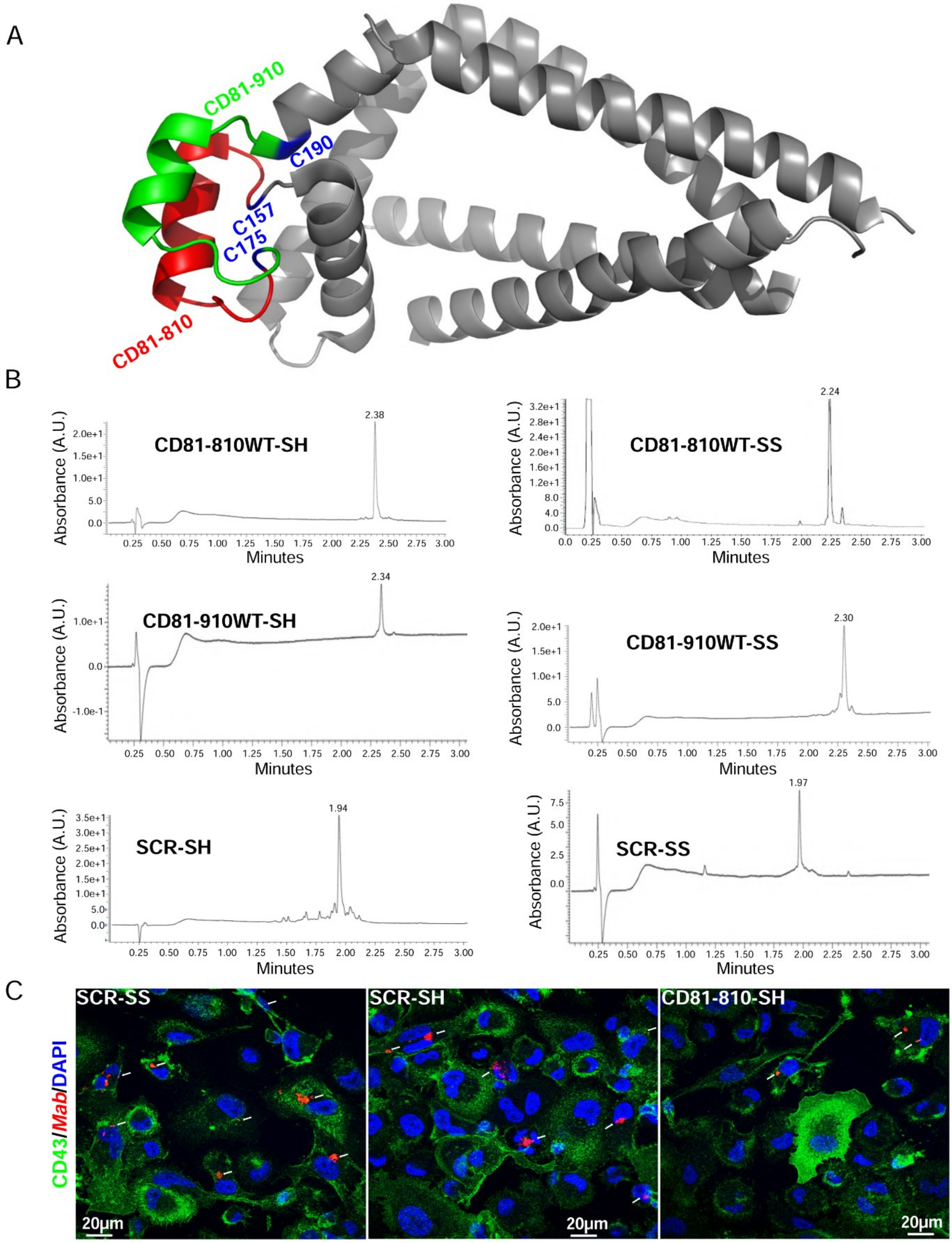

Supplementary Figure 3 - Positioning, purity and activity of CD81-LEL-derived peptides, Related to Figure 2

(A) Visualization of the CD81-810 and CD81-910 peptides in the human CD81 protein structure. Cartoon representation of the CD81 protein (PDB 5TCX, in grey) visualized using PyMol Viewer. The peptides used in this study are in red (CD81-810, C_{157}GSSTLTALTTSVLKNNLC_{175}) and in green (CD81-

910, C_{175}PSGSNIISNLFKEDC$_{190}$). The cysteine residues used for the cyclization (C157-C175 for CD81-810 and C175-C190 for CD81-910) are indicated in blue.

(B) HPLC profile of the different synthetic peptides from CD81-LEL. The chromatographic profile (UV at 214 nm) shows main peaks with a high percentage (>85%) of purity. Corresponding mass spectrometry analysis of the main peak corresponds to the expected mass.

(C) Three immunofluorescent fields were collected with a confocal microscope (40 x magnification) 3 h after infection, revealing macrophages infected with *Mab* after pre-treatment with the 810-derived or scrambled (SRC) peptides. The nuclei are blue, and the CD43 protein linked with macrophage plasma membrane is green. *Mab* (in red) inside macrophages are indicated by white arrows.

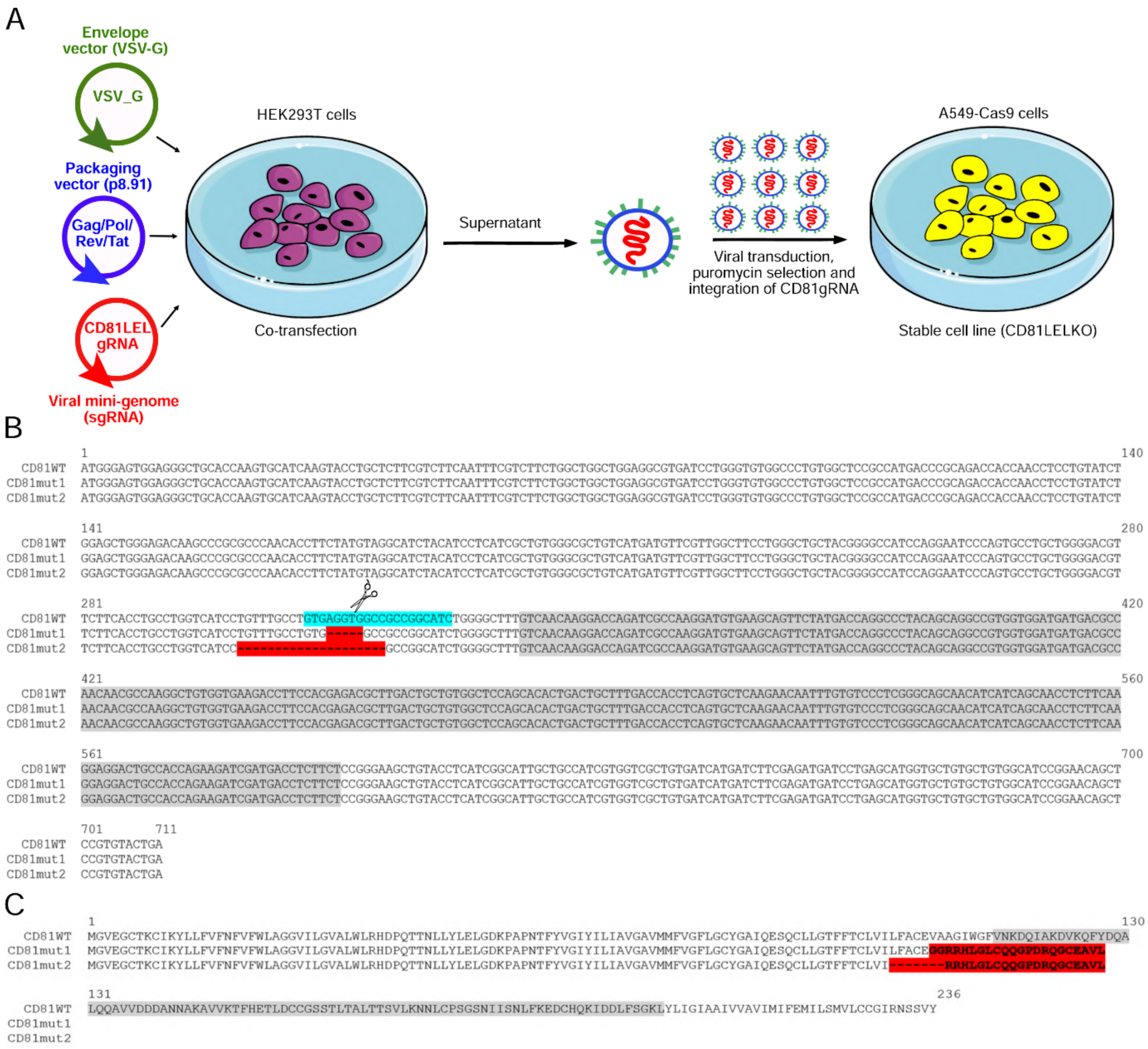

Supplementary Figure 4 – Strategy, disruption and generation of CD81-LEL knock-out cells, Related to Figure 3

(A) Overview of the Lentivirus CRISPR/Cas9 system used to disrupt the LEL of CD81 in A549 cells. In this system, a lentivirus is generated from HEK293T cells encoding for expression of the CD81-LEL sgRNA under the U6 promoter. The process of generating an infectious transgenic lentivirus is outlined in this schematic. Three plasmids are transfected into HEK293T cells: a transfer plasmid (red), a packaging plasmid (blue) and an envelope plasmid (green). After incubation, the virus-containing supernatant is collected, filtered and then used to transduce A549 cells. Briefly, A549 target cells are transduced with the lentivirus expressing *Cd81-LEL* sgRNA and selected for integration using puromycin. The final result is a mutant cell line (A549-CD81-LEL-KO) stably expressing both Cas9 (selection by blasticidin) and *Cd81-LEL* sgRNA (selection by puromycin).

(B) The gRNA sequence (blue) consisting of a 20-nucleotides fragments pairs with the target DNA located upstream of a requisite 5'-NGG adjacent motif (PAM) and the *LEL* sequence (grey). Cas9 will break the double-stranded DNA (scissors symbol) at exon 4 located upstream of the sequence that

codes for the LEL region. CRISPR/cas9 DNA cleavage and NHEJ repair that resulted in two different multi-base deletions were detected after we amplified the open reading frame of the *Cd81* gene by PCR, followed by multiple sequencing reactions of several amplicons to accurately determine the suppressed bases. The deletion regions are shown in red.

(C) The frame-shift generated a shorter CD81 protein lacking its LEL (grey) and terminating in an erroneous peptide sequence (in red) that does not exist in CD81 expressed by normal cells.

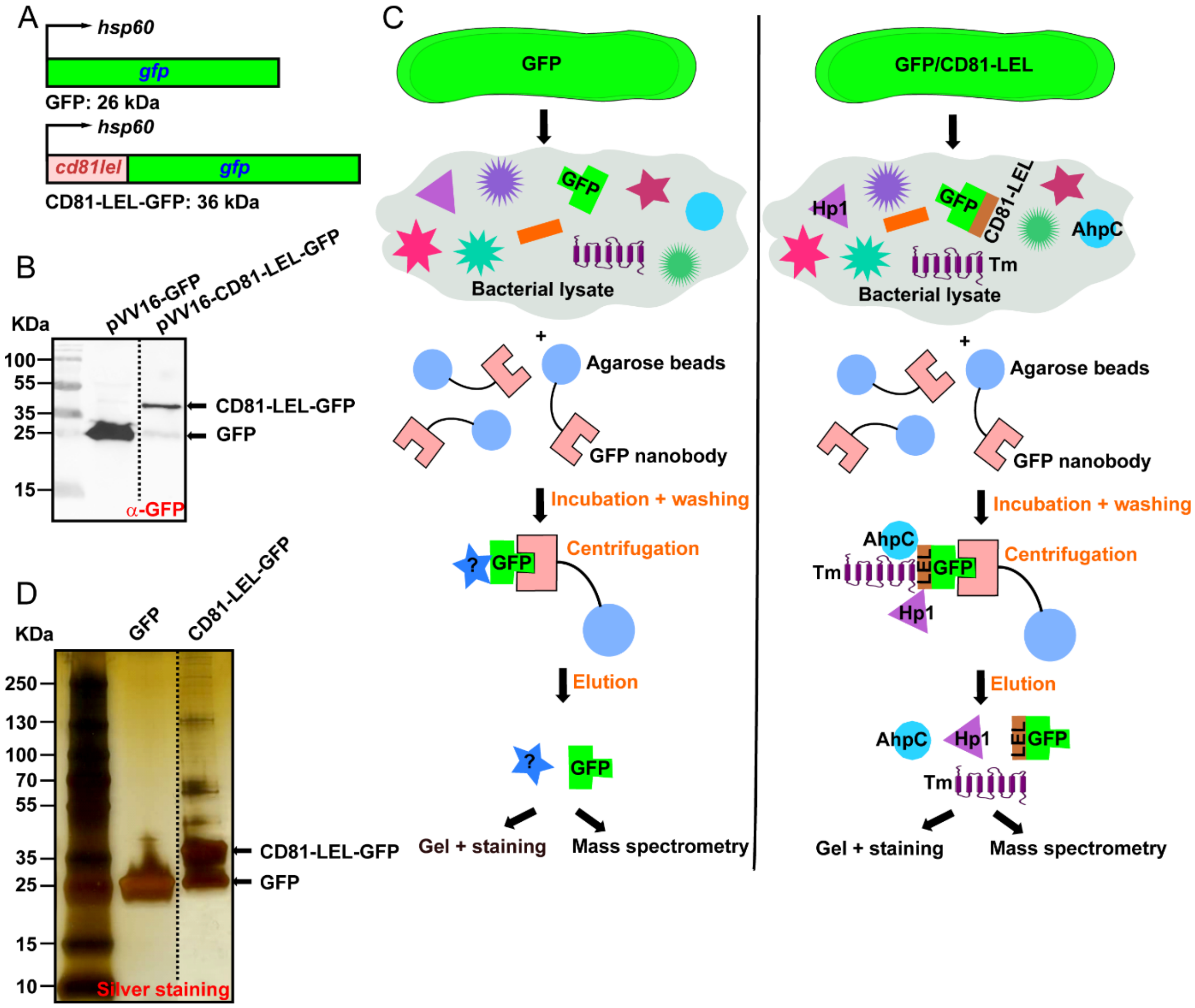

E

Protein name	Accession numbers	Molecular weight (kDa)	Total number of unique peptides	
			GFP pull-down	CD81-LEL-GFP pull-down
**Hsp16.7**	MAB_3467c	16.7	0	5
**AhpC**	MAB_4408c	21.6	0	11
**Tm**	MAB_3498c	107.2	0	5
**Hp1**	MAB_1957	13	0	5

Supplementary Figure 5 – Identification of CD81-LEL-interacting partners by GFP-pulldown and mass spectrometry, Related to Figure 4

(A) CD81-LEL was fused to a C-terminal GFP tag under the control of the *hsp60* promoter. Molecular weight in kDa is indicated at the bottom of each schema.

(B) Western blotting of *Mab* strains lysates expressing GFP or CD81-LEL-GFP fusion protein using anti-GFP antibodies.

(C) Illustration of the GFP nanobody pull-down approach.

(D) Silver-stained gel showing the eluted protein profile that were either bound to GFP or to CD81-LEL, and detected after immunoprecipitation using anti-GFP nanobodies. The bacterial strain stably

expressing GFP was used as a negative control. The eluates of GFP and CD81-LEL-GFP beads were subjected to mass spectrometry.

(E) Selection of the most representative CD81-LEL protein interactants.

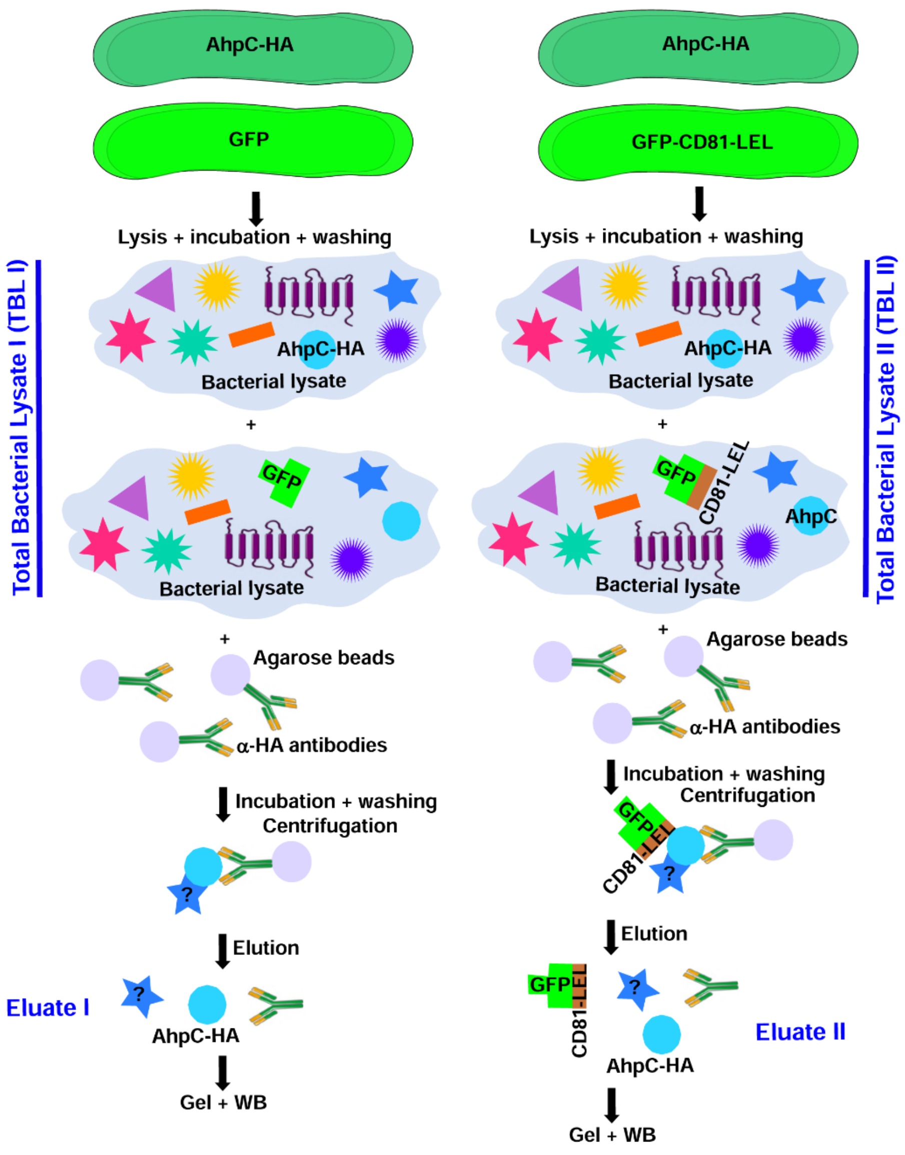

Supplementary Figure 6 – Schematic illustrating the procedure we used to validate the AhpC/ CD81-LEL interaction, Related to Figure 4

A *Mab* strain overproducing Ha-tagged AhpC was created. To validate the interaction between CD81-LEL-GFP and AhpC-HA, bacteria co-expressing the receptor (CD81-LEL-GFP) and the ligand (AhpC-HA) were lysed and the corresponding supernatants were mixed together (bacterial lysate number II). As a negative control, the bacterial lysate corresponding to bacteria expressing GFP alone was mixed with the one expressing AhpC-HA (bacterial lysate number I). Lysates I and II were then incubated with beads coated with anti-HA antibodies. After several washes, eluted proteins were separated by SDS-PAGE and Western blotting was performed using anti-HA or anti-GFP antibodies to validate the interaction between the bait protein (AhpC-HA ligand) and the prey (CD81-LEL-GFP receptor).

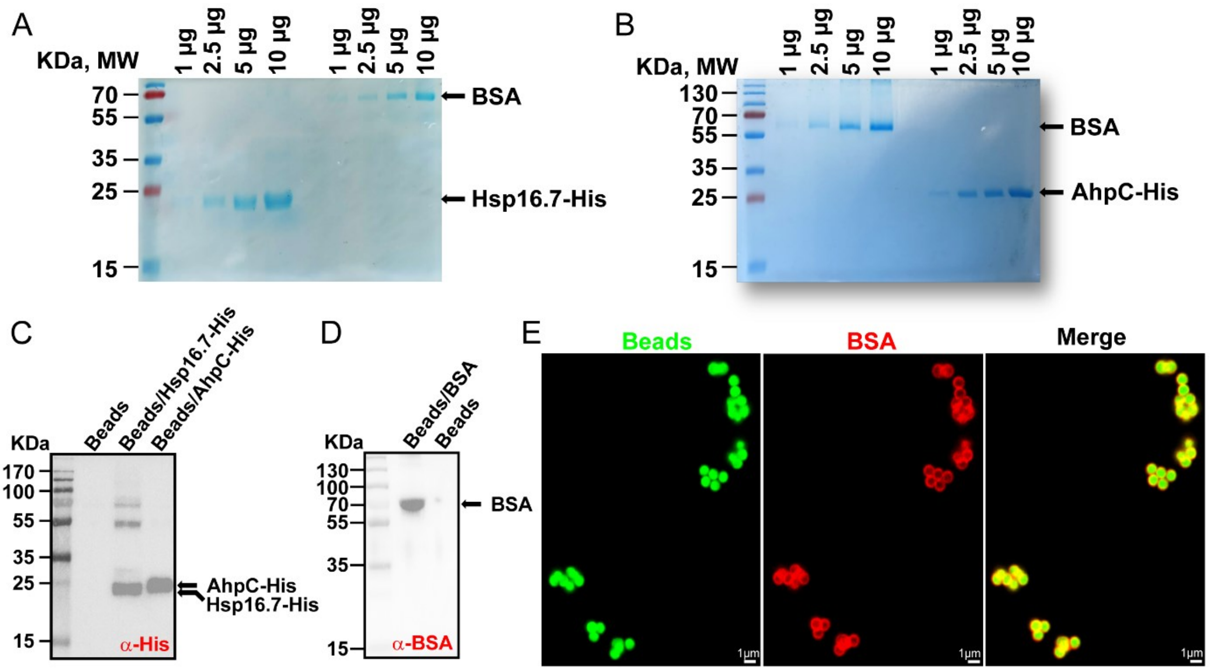

Supplementary Figure 7 – Purity of the recombinant proteins and bead coating, Related to Figure 5

(A and B) SDS-PAGE and Coomassie Blue staining of purified recombinant Hsp16.7-His and AhpC-His proteins. BSA standards, 1 µg (lanes 2 and 7), 2.5 µg (lanes 3 and 8), 5 µg (lanes 4 and 9), and 10 µg (lanes 5 and 10); molecular weight standard in kDa on the left (lanes 1).

(C and D) Western blotting of Hsp16.7-His, AhpC-His and BSA proteins conjugated to fluorescein beads. Non-conjugated beads (indicated by B) were used as negative control. Anti-His and anti-BSA antibodies were used to detect Hsp16.7 and AhpC (D) or BSA (E). Molecular weight marker is shown on the left.

(E) BSA protein on the surface of fluorescein-tagged latex beads was detected using rabbit anti-BSA antibodies and Alexa Fluor 594 conjugated goat anti-rabbit secondary antibodies.

Supplementary Table 1. Primers used in this study. Restriction sites are in bold and underlined - Related to STAR Methods

Number	5'→3' sequence	Restriction site	F (sense), R (antisense)
**Cloning in pUX1-*katG***			
1	gaga**<u>TTAATTAA</u>**GACACGAGTCGGGTAAGCAGTTC	PacI	To clone the left arm of *ahpC* in pUX1-*katG* (F)
2	gaga**<u>CAATTG</u>**GATGGTGCGCAGAGTCACGATTC	MfeI	To clone the left arm of *ahpC* in pUX1-*katG* (R)
3	gaga**<u>GAATTC</u>**AGCGCAGGCGTCTAGGAGG	EcoRI	To clone the right arm of *ahpC* in pUX1-*katG* (F)
4	gaga**<u>GCTAGC</u>**AAAGAGCAGCGTGGGCTCTTTGG	NheI	To clone the right arm of *ahpC* in pUX1-*katG* (R)
**Primers to verify double homologous recombination**			
5	TATCAGCTTCGGTCCGTTCTTTCAGAG (1)	-	To check double homologous recombination Δ*ahpC* (F)
6	CATCGGGACAGTACTAAAGAACATCAAGAG (4)	-	To check double homologous recombination Δ*ahpC* (R)
7	GAAATGGCGTTTACGTGCGATAGC (2)	-	To check double homologous recombination Δ*ahpC* (F)
8	GAGCTTGAGGTCCTTGGCGTACT (3)	-	To check double homologous recombination Δ*ahpC* (R)
**Cloning in pMV306-HygR**			
9	TACCCATACGATGTTCCAGATTACG	-	Primers to amplify the entire backbone of the pMV306 vector (F)

10	CATGCGGTTGTGAGCGCTCAC	-	Primers to amplify the entire backbone of the pMV306 vector (R)
11	CGCTCACAACCGCATGactctgcgcaccatcggc	-	To clone the *ahpC-ha* ORF in pMV306 (F)
12	GGAACATCGTATGGGTAgacgcctgcgctcatcagct	-	To clone the *ahpC-ha* ORF in pMV306 (R)
**Cloning in pMV261-KanR**			
13	gaga**AAGCTT**CCGTGACTCTGCGCACCATCGG	HindIII	To clone the *ahpC-HA* ORF in pMV261 (F)
14	gaga**GTTAAC**CTAAGCGTAATCTGGAACATCGTAT<br>GGGTAGACGCCTGCGCTCATCAGCTC	HpaI	To clone the *ahpC-HA* ORF in pMV261 (R)
15	gagaga**TGGCCA**CCGTGCAGAGGTCGCGAGACTG	MscI	To clone the *fmt-HA* ORF in pMV261 (F)
16	gaga**GAATTC**CTAAGCGTAATCTGGAACATCGTAT<br>GGGTAGGCCTTGACAAAGCGGTACATCC	EcoRI	To clone the *fmt-HA* ORF in pMV261 (R)
**Cloning in pVV16-KanR**			
17	gaga**catatg**GTCAACAAGGACCAGATCGCCA	NdeI	To clone the *CD81-LEL* in pVV16 (F)
18	gaga**ggatcc**CAGCTTCCCGGAGAAGAGGTCA	BamHI	To clone the *CD81-LEL* in pVV16 (R)
**Cloning in pGEX-2T-AmpR**			
19	gaga**ggatcc**ccggGAATTAACAAGGACCAGAT	BamHI	To clone the *CD81-LEL* in pGEX-2T (F)
20	gaga**GAATTC**tcagtcacgatGAATTACAGCTTCCCG	EcoRI	To clone the *CD81-LEL* in pGEX-2T (R)
21	gaga**AGATCT**GCCTACTACCAGCAGCTGAACACG	BglII	To clone the *CD151-LEL* in pGEX-2T (F)
22	gaga**GAATTC**CCGGCAGGTGCTCCTGGATGAAGGTCTC	EcoRI	To clone the *CD151-LEL* in pGEX-2T (R)
**Cloning in pET30-KanR**			
23	gaga**gaattc**CTAGGCACTGATTTTCCCTCTTGCGGAA	EcoRI	To clone the *hsp16.7* ORF in pET30 (F)
24	gaga**GGTACC**ATGGAGAACCTGTACTTCCA<br>GGGTATGCTGATGCGTACCG	KpnI	To clone the *hsp16.7* ORF in pET30 (R)

25	gaga**<u>gaattc</u>**CTAGACGCCTGCGCTCATCAG	EcoRI	To clone the *ahpC* ORF in pET30 (F)
26	gaga**<u>GGTACC</u>**ATGGAGAACCTGTACTTCCAGGGT GTGACTCTGCGCACCATCGGCGATGAGTTCC	KpnI	To clone the *ahpC* ORF in pET30 (R)
**Cloning in LentiGuidePuro-AmpR**			
27	CACCGGATGCCGGCGGCCACCTCAC	BsmBI	To clone the *CD81-LEL* sgRNA in LentiGuide-Puro vector (F)
28	AAACGTGAGGTGGCCGCCGGCATCC	BsmBI	To clone the *CD81-LEL* sgRNA in LentiGuide-Puro vector (R)
